# Effect of remote ischemic preconditioning, nicorandil, and trimetazidine in contrast-induced nephropathy: a network meta-analysis of randomized controlled trials

**DOI:** 10.1080/0886022X.2024.2431141

**Published:** 2024-11-27

**Authors:** Hanchao Gao, Weilong Li, Chuanchuan Sun, Shiping Zhu, Fanna Liu, Xinhai Zhao, Shaodong Luan, Shengyun Sun, Yeye Yu

**Affiliations:** a Department of Nephrology, Shenzhen Longhua District Central Hospital, Shenzhen Longhua District Key Laboratory for Diagnosis and Treatment of Chronic Kidney Disease, Shenzhen, China; b Department of Nephrology, The First Affiliated Hospital of Jinan University, Guangzhou, China; c Department of Chinese Traditional Medicine, The First Affiliated Hospital of Jinan University, Guangzhou, China

**Keywords:** Contrast-induced nephropathy, remote ischemic preconditioning, nicorandil, trimetazidine, network meta-analysis

## Abstract

**Introduction:**

Contrast-induced nephropathy (CIN) is a potential complication associated with the administration of intravenous contrast agents. The objective of this study was to evaluate the effectiveness of remote ischemic preconditioning (RIPC) and two pharmacological interventions in preventing CIN.

**Methods:**

Randomized controlled trials (RCTs) examining the efficacy of RIPC, nicorandil, and trimetazidine in treating CIN were searched within databases such as PubMed, Cochrane Library, Embase, and Web of Science. The primary outcome was the incidence of CIN. The consistency model was used to address heterogeneity and enhance model fit. The assessment of consistency between direct and indirect evidence was conducted through the node-splitting method. Posterior probability estimates and surface under the cumulative ranking area (SUCRA) ranked interventions based on their effectiveness in preventing CIN. The Grading of Recommendations, Assessment, Development, and Evaluations (GRADE) framework was used to grade the quality of evidence.

**Results:**

Based on hydration therapy, RIPC, nicorandil, and trimetazidine all showed prophylactic effects on CIN compared to control groups. The SUCRA results showed that RIPC (SUCRA = 37.7%, PrBest = 0.4%), nicorandil (SUCRA = 91.2%, PrBest = 74.7%), and trimetazidine (SUCRA = 71.0%, PrBest = 24.9%). However, there were no significant differences between the nicorandil, RIPC, and trimetazidine groups. Subgroup analysis suggested that there was still a protective effect in populations with mean estimated glomerular filtration rate (eGFR) less than 60 mL/min/1.73 m^2^ or with a high prevalence of diabetes mellitus.

**Conclusions:**

Nicorandil, trimetazidine, and RIPC all showed renal protective effects. Based on hydration, nicorandil, trimetazidine, and RIPC may show better prophylaxis against CIN than hydration alone after intravenous contrast administration.

## 1. Introduction

Contrast-induced nephropathy (CIN) is a severe complication that occurs during radiologic procedures with exposure to intravenous contrast media. Some studies have called it contrast-induced acute kidney injury (CI-AKI). Clinical radiologic procedures using contrast agents include coronary angiography (CA), percutaneous coronary intervention (PCI), enhanced CT examination, and transcatheter aortic valve implantation (TF-TAVI). Common types of acute kidney injury in hospitalized patients include CIN. Advanced age, abnormal renal function, anemia, and heart failure are risk factors for CIN [1]. Renal ischemia–reperfusion injury plays a vital role in the development of CIN [2]. Preventive measures are still the mainstay of CIN, and the more accepted prophylactic measure is hydration. Other ­measures reported to be potentially effective include remote ischemic preconditioning (RIPC), nicorandil, trimetazidine, and N-acetylcysteine, but their effectiveness is controversial.

RIPC is done by blocking blood flow to non-target tissues, for instance, blood vessels or limbs, to induce a short period of intermittent ischemia–reperfusion before the ischemia–reperfusion injury. RIPC acts by stimulating endogenous protective mechanisms. RIPC is a noninvasive prophylactic measure. Various studies have used it before cardiac surgery [3] and non-cardiac surgery [4] to explore whether it protects against acute kidney injury and all-cause mortality or prevents contrast nephropathy before intravenous application of contrast media [5]. Some studies reported a protective effect against CIN [6,7], some considered it effective in intermediate and high-risk populations, and others did not find a renal protective effect [8,9].

Because the pathogenesis of CIN may be related to renal ischemia, recent studies have shown that drugs with anti-vasoconstrictive or anti-ischemic effects may have a preventive impact on CIN, such as nicorandil [10] and trimetazidine [11]. Nicorandil is a common clinical drug with potassium channel-opening properties, which is thought to improve blood circulation [10]. Many randomized controlled trials (RCTs) have explored whether intravenous or oral nicorandil could prevent CIN before and after radiologic procedures. Some studies have found that prophylaxis with nicorandil is effective in preventing CIN in patients with type 2 diabetes [12] or kidney disease [13] who were undergoing CA. However, other studies have concluded that intravenous nicorandil has no significant role in preventing CIN after PCI in diabetic patients with acute coronary syndromes [14]. Trimetazidine is believed to maintain intracellular levels of phosphocreatine and ATP, alleviating cellular acidosis and free radical damage [15]. It has been shown that trimetazidine prevents the development of CIN in people with kidney disease after using contrast media and protects the renal function of patients [16,17]. Taken together, these studies suggested that the role of RIPC, nicorandil, and trimetazidine on renal function after radiological procedures remains elusive.

To clarify the above issues, this study conducted a network meta-analysis (NMA) of clinical trials concerned with the role of RIPC, nicorandil, and trimetazidine on renal function after CA, PCI, or other radiological procedures with intravenous contrast agents on post-procedural CIN, requirement for renal replacement therapy and survival.

## 2. Methods

### 2.1. Protocol and registration

The protocol for this NMA was registered in PROSPERO (CRD42024538454). The PRISMA (Preferred Reporting Items for Systematic Reviews and Meta-Analysis) guidelines were followed when conducting this systematic review and meta-analysis.

*Search strategy*:

Published RCTs related to CIN therapies were searched in PubMed, the Cochrane Library, Web of Science, and Embase. Search terms include remote ischemic preconditioning, nicorandil, trimetazidine, contrast medium, angiography, and RCTs. Details of the search strategy are recorded in the Supplementary Material.

*Inclusion criteria*:

1. The subject of the study accepted a radiological procedure that required contrast media;
2. Interventions including RIPC, nicorandil, and trimetazidine; both experimental and control groups received hydration;
3. Outcome included the incidence of CIN or CI-AKI;
4. Full text and data could be found;
5. The study type was RCT.

*Exclusion criteria*:

1. Questionable data;
2. There was a significant difference when comparing the baseline renal function of patients in the experimental and control groups;
3. Repeatedly published data;
4. Any other items that do not meet the inclusion criteria.

### 2.2. Study outcomes

The incidence of CIN or CI-AKI is the primary outcome, as defined in the protocol of the original RCT. Criteria for CIN determination include:

1. *European Society of Urologic Radiology (ESUR) criteria*: A rise of more than 25% or 44 μmol/L in serum ­creatinine (SCr) within three days of intravenous contrast injection without other etiologic factors [18].
2. *Acute Kidney Injury Network (AKIN)*: A rise in absolute SCr level of ≥0.3 mg/dL (26.4 μmol/L) or a rise in SCr level of more than 50% from baseline or a decrease in urine volume (documented oliguria of <0.5 mL/kg/h for >6 hours) within 48 hours [19].
3. *CI-AKI based on liver-type fatty acid-binding protein (L-FABP)*: L-FABP >17.4 µg/g Cr or an increase in L-FABP >25% from baseline provided baseline L-FABP was >17.4 µg/g Cr within 24 h [20,21].

The reason for using an L-FABP-based definition of CIN was that studies have found that urinary L-FABP levels increased rapidly and peaked within 24 h of contrast administration, which was thought to reflect tubulointerstitial injury, such as ischemic injury [20,22].

Other outcomes include the impact of interventions on hospitalization, hemodialysis, and mortality rates. Analyze when there is enough data.

Subgroup analysis was conducted on the incidence of CIN in population with different mean estimated glomerular filtration rate (eGFR) levels or varying proportions of diabetes.

### 2.3. Data collection and quality assessment

A uniform standardized data extraction form was designed and two independent reviewers (HCG and YYY) extracted the following data.

1. *Study general characteristics*: First author, year of publication, language, region, duration, type of study.
2. *Patient characteristics*: Primary eligibility and excluded criteria, age, sex, percentage of patients with diabetes and hypertension, type of intervention, types of contrast media, and contrast medium dose.
3. *Intervention characteristics*: Type, dose, and timing.
4. *Control group*: Type, dose, and timing.
5. *Definition of CIN*: AKIN OR KDIGO classification, other definitions, or not reported.
6. *Results*: For binary outcomes, the data collected included the number of events and total patients analyzed. For continuous outcomes, the data collected included the mean, standard deviation, and number of patients.

Under the guidance of the RCT Risk of Bias tool in the Cochrane Handbook, two reviewers (HCG and YYY) independently assessed the risk of bias for each trial.

### 2.4. Consistency checks, publication bias assessment, and sensitivity checks

The fundamental assumption of NMA is that direct and indirect evidence estimate the same parameters. Inconsistency means that direct and indirect comparisons conflict. The node-splitting method evaluated consistency between the direct and indirect evidence. Comparisons were considered inconsistent when *p* < .05. To reduce heterogeneity and improve model fit, this NMA used a consistency model [23]. Funnel plot analysis was performed for all network comparisons to assess symmetry and publication bias. Harbord test was used to assess publication bias quantitatively. Sensitivity checks are performed by eliminating comparisons where the risk of sensitivity may be high. Sensitivity checks are performed when there are sufficient data or when the funnel plot shows significant deviations.

### 2.5. Data synthesis and analysis

**Figure 1.**
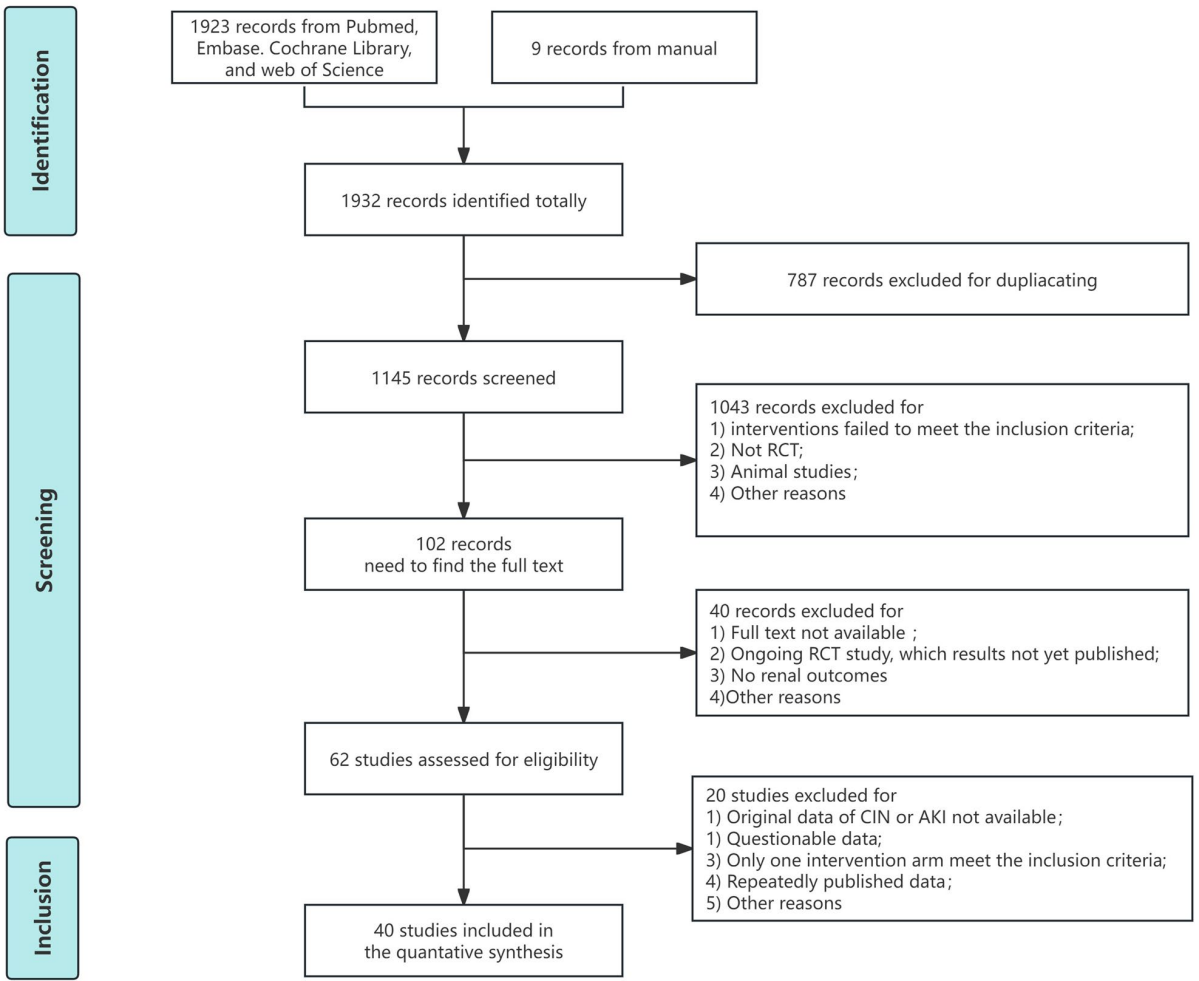
Flow diagram of the network meta-analysis. Published studies before 9 May 2024 were searched using a standardized strategy. One thousand nine hundred and thirty-two records were identified from the databases. According to the inclusion and exclusion, 40 publications were eventually included in the network meta-analysis. RCT: randomized controlled trial; CIN: contrast-induced nephropathy.

A network of publications was designed to use RIPC, nicorandil, and trimetazidine to prevent CIN. Figure 1 illustrates the flowchart for the NMA. The meta-aggregation was performed by Markov Chain Monte Carlo simulation chains in the Bayesian-based framework. Odds ratios (ORs) and 95% confidence intervals (95% Crl) were estimated for binary outcomes, as well as mean difference (MD) and 95% 95% Crl for continuous outcomes. The obtained posterior probabilities and the preferred probability ranking curve (surface under the cumulative ranking area, SUCRA) were used to rank the protective effects of the interventions involved in the comparison. Larger values of SUCRA suggest a better effect. If there were differences between the interventions, the comparisons were distinguished as superior or inferior. We assessed the heterogeneity across trials by the *I*^2^. The funnel plot assessed publication bias. The Harbord test was used to assess publication bias quantitatively. R 4.3.3, Review Manager version 5.4, and State 17 were applied for data analysis and figure formation. *p* < .05 was considered statistically significant.

### 2.6. Certainty of evidence

The GRADE (Grading of Recommendations, Assessment, Development, and Evaluations) framework was used to evaluate the certainty of the evidence for the outcome of CIN. Two independent reviewers (LWL and YYY) completed this assessment.

## 3. Results

### 3.1. Identified publications

**Figure 2.**
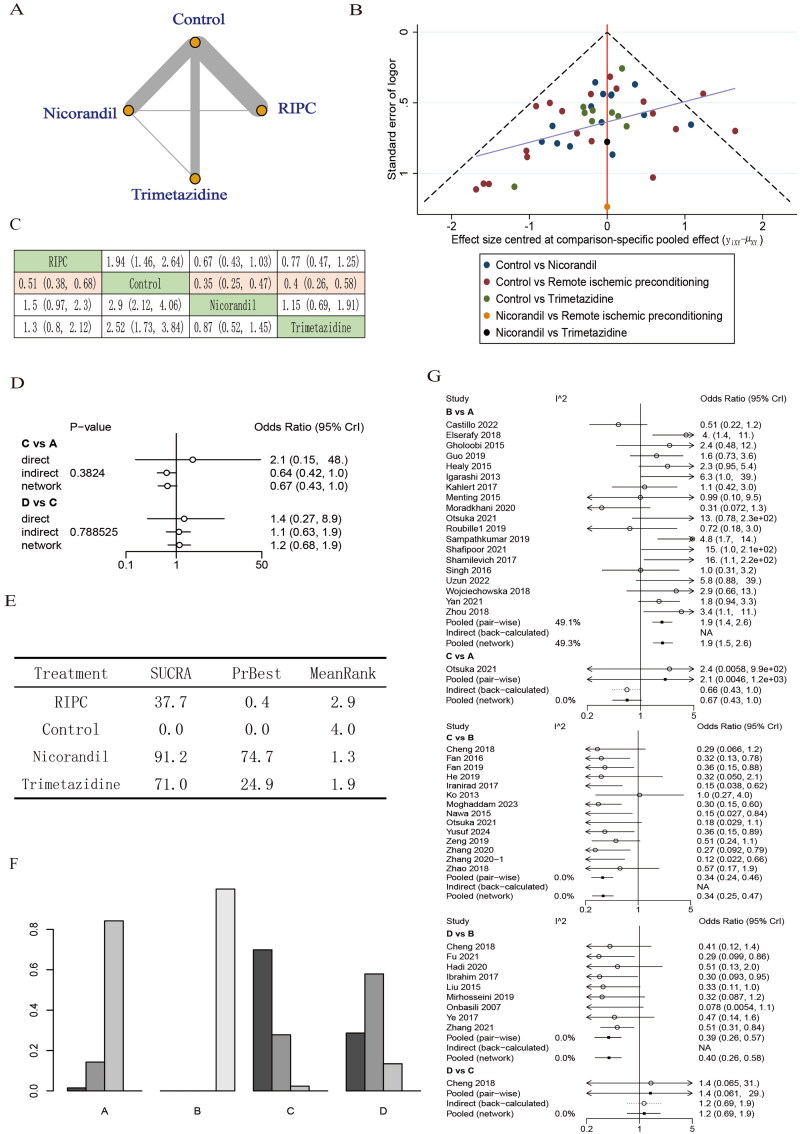
Effect of RIPC, nicorandil, and trimetazidine on CIN occurrence. (A) Network maps present all interventions with direct comparisons. (B) Funnel plots. The dots represent included studies and the symmetry of the funnel plot was used to assess publication bias. (C) League tables presenting head-to-head comparisons of the incidence of CIN. Comparisons of statistical significance were marked pink. (D) Consistency evaluation shows the consistency of direct and indirect comparison, incidence of CIN presented as odds ratio (OR) and 95% Crl. *p* < .05 was considered statistically significant, and inconsistency is deemed to exist of every individual intervention compared with every other presented in this figure. (E) The SUCRA results show the probability sequence of RIPC, nicorandil, and trimetazidine in reducing CIN. (F) Ranking graphs. The darker the color of the bar, the higher the order. (G) Heterogeneity test. Lines represent the efficacy estimate with ln-values (values including OR and its 95% Crl). A: RIPC; B: control; C: nicorandil; D: trimetazidine; Crl: credible interval.

Published publications before 9 May 2024 were searched using a standardized strategy. Ultimately, a total of 40 RCTs [6–14,16,17,21,24–51] with 7219 patients were included, and treatment measures included RIPC, nicorandil, and trimetazidine. A comparative network diagram of CIN is shown in Figure 2(A). Among all the studies, two were three-arm designs (one was RIPC, nicorandil, and control [36]; another was nicorandil, trimetazidine, and control [46]). All studies reported the occurrence of CIN or CI-AKI. There were no differences in basic characteristics such as sex percentage, age, percentage of patients with diabetes mellitus, baseline creatinine, and contrast dose between the intervention and control groups in the included studies. All patients received hydration (intravenous normal saline (0.5–1.5 mL/kg/h)) from 2 to 12 h before to 6–24 h after the procedure, adjusting maintenance time and speed appropriately according to left ventricular ejection fraction (LEVF). Characteristics and baseline information of the enrolled publications were summarized in Supplementary Table S1.

### 3.2. Quality assessment, sensitivity checks, publication bias check, and model assessment

For the primary outcome, the majority of trials have elements that may be unclear; some were low risk of bias, but a few have a high risk of bias (Supplementary Figures S1 and S2). For the secondary endpoints, a few trials have a high risk of bias (Supplementary Figure S3–S6). Sensitivity checks suggested stable outcome indicators (Supplementary Figures S7–S9). According to the funnel plot, the two sides were not perfectly symmetrical, and two studies were outside the funnel plot confidence intervals (Figure 2(B)). Harbord test was used to assess publication bias quantitatively. The results suggested that none of them had publication bias (*p* > .05) (Supplementary Table S2). Trajectory density graphs were used to conduct model assessment. The results show the model’s fair degree of convergence and fit (Supplementary Figures S10–S16).

### 3.3. Main outcomes

Based on hydration therapy, RIPC (ORs = 0.51, 95% Crl = (0.38, 0.68)), nicorandil (ORs = 0.35, 95% Crl = (0.25, 0.47)), and trimetazidine (ORs = 0.51, 95% Crl = (0.26, 0.58)) all showed prophylactic effects on CIN compared to control groups accepting hydration therapy only according to the network maps and league tables (Figure 2(A,C)). Consistency models were used to test the stability of the results, and the results show good consistency (*p* > .05) **(**Figure 2(D)**)**. The SUCRA results showed that RIPC (SUCRA = 37.7%, PrBest = 0.4%), nicorandil (SUCRA = 91.2%, PrBest = 74.7%), and trimetazidine (SUCRA = 71.0%, PrBest = 24.9%). Using the obtained posterior probability and SUCRA to rank all interventions, nicorandil ranked 1st, trimetazidine ranked 2nd, and RIPC ranked 3rd, all higher than controls in preventing CIN (Figure 2(E,F)). However, there were no significant differences between the nicorandil, RIPC, and trimetazidine groups. Heterogeneity among trials was low for the overall analysis of the incidence of CIN **(**Figure 2(G), Supplementary Table S3).

### 3.4. Subgroup analysis

#### 3.4.1. eGFR

**Figure 3.**
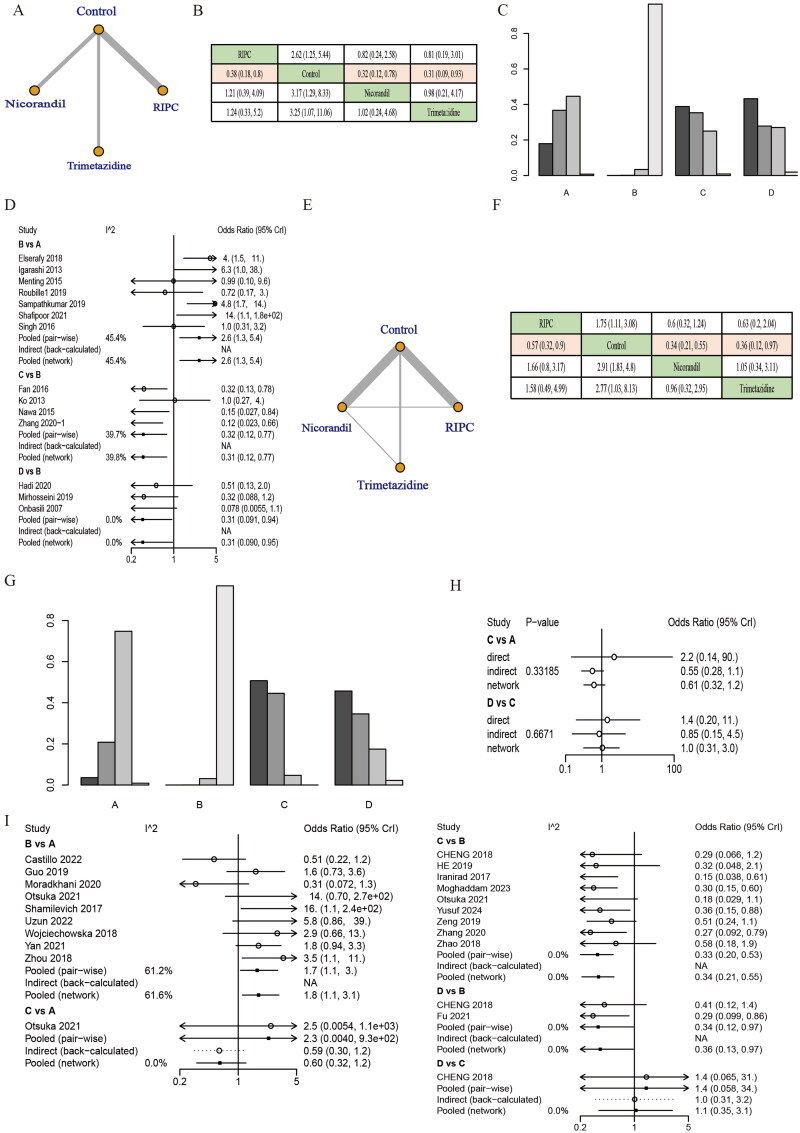
Subgroup analysis based on mean eGFR levels of the effect of RIPC, nicorandil, and trimetazidine on the incidence of CIN. (A–D) Among the population with mean eGFR <60 mL/min/1.73 m^2^. (A) Network maps, (B) league tables, efficacy estimates with significant differences are in pink. (C) Ranking graphs, (D) heterogeneity test. (E–I) Among the population with mean eGFR >60 mL/min/1.73 m^2^. (E) Network maps, (F) league tables, (G) ranking graphs, (H) consistency evaluation, and (I) heterogeneity test. A: RIPC; B: control; C: nicorandil; D: trimetazidine.

According to the network maps and league tables, RIPC, nicorandil, and trimetazidine showed protective effects on CIN compared to controls among the population with mean eGFR < 60 mL/min/1.73 m^2^. However, there were no significant differences between the nicorandil, RIPC, and trimetazidine groups (Figure 3(A–C)). Heterogeneity among trials was low in the population with mean eGFR < 60 mL/min/1.73 m^2^ (Figure 3(D), Supplementary Table S4). In addition, RIPC, nicorandil, and trimetazidine also showed protective effects on CIN compared to control groups among the population with mean eGFR > 60 mL/min/1.73 m^2^. Although nicorandil ranked 1st, there were no significant differences between these three treatments (Figure 3(E–G)). The consistency check result showed there was no inconsistency (*p* > .05) (Figure 3(H)). Similarly, heterogeneity among trials was low in the population with mean eGFR > 60 mL/min/1.73 m^2^ (Figure 3(I), Supplementary Table S5).

#### 3.4.2. Diabetes

**Figure 4.**
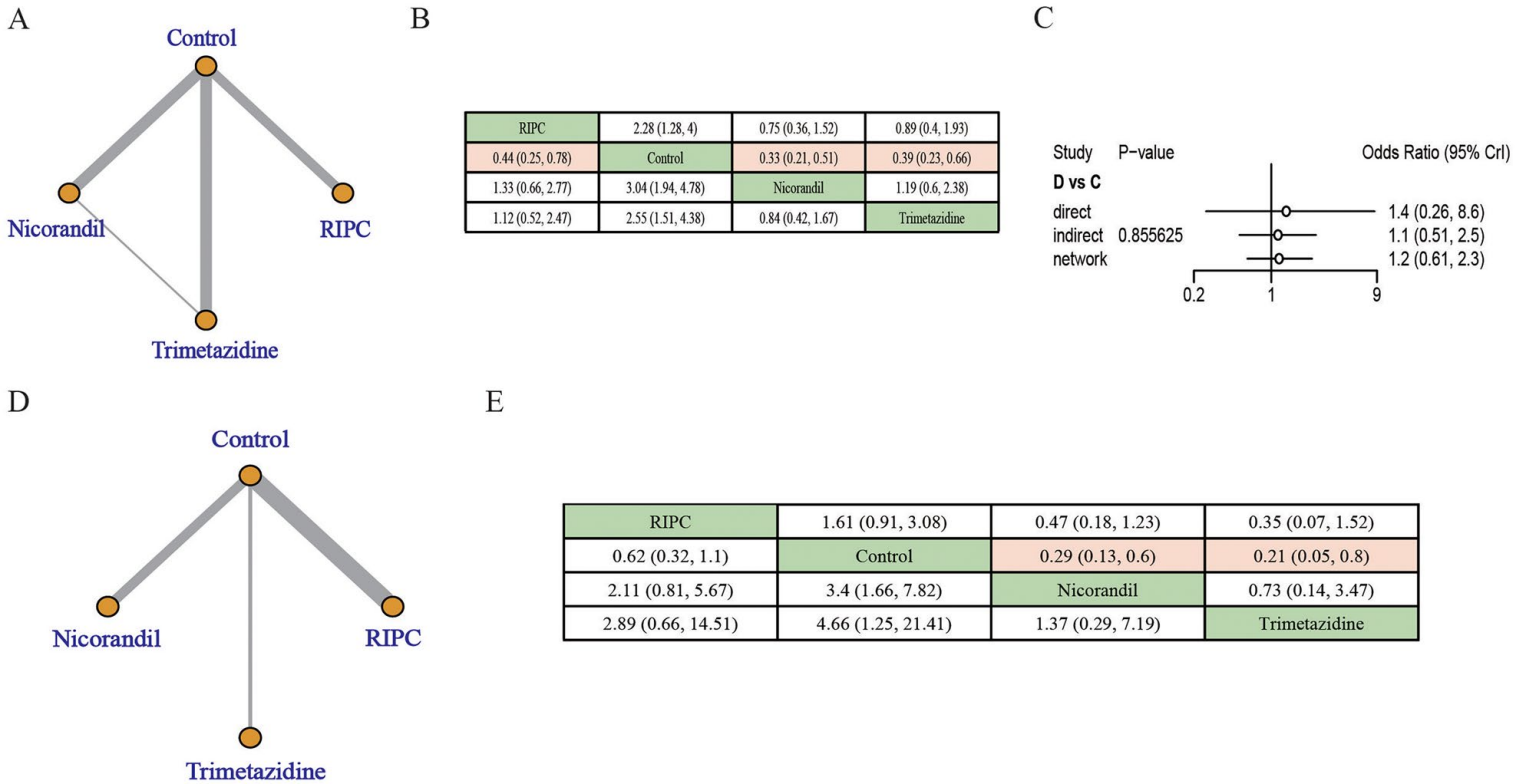
Subgroup analysis based on proportion of patients with diabetes of the effect of RIPC, nicorandil, and trimetazidine on the incidence of CIN. (A–C) In populations with >50% diabetes. (A) Network maps, (B) league tables, efficacy estimates with significant differences are in pink. (C) Consistency evaluation. (D, E) In populations with <50% diabetes. (D) Network maps, (E) league tables. Efficacy estimates with significant differences are in pink. A: RIPC; B: control; C: nicorandil; D: trimetazidine.

Among trials with more diabetic patients, RIPC, nicorandil, and trimetazidine also showed protective effects on CIN compared to controls. However, there were no significant differences between the nicorandil, RIPC, and trimetazidine groups (Figure 4(A,B)). Consistency check results showed good consistency (*p* > .05) (Figure 4(C)). Among trials with fewer diabetic patients, only nicorandil and trimetazidine showed protective effects on CIN compared to controls, and there were no significant differences between the nicorandil and trimetazidine groups (Figure 4(D,E)). Heterogeneity among trials was low in the subgroup analysis of CIN occurrence in populations with >50% diabetes. However, there was some heterogeneity among trials in populations with <50% diabetes, and the comparison of RIPC versus controls presented a high degree of heterogeneity (Supplementary Tables S6 and S7).

### 3.5. The secondary endpoints

**Figure 5.**
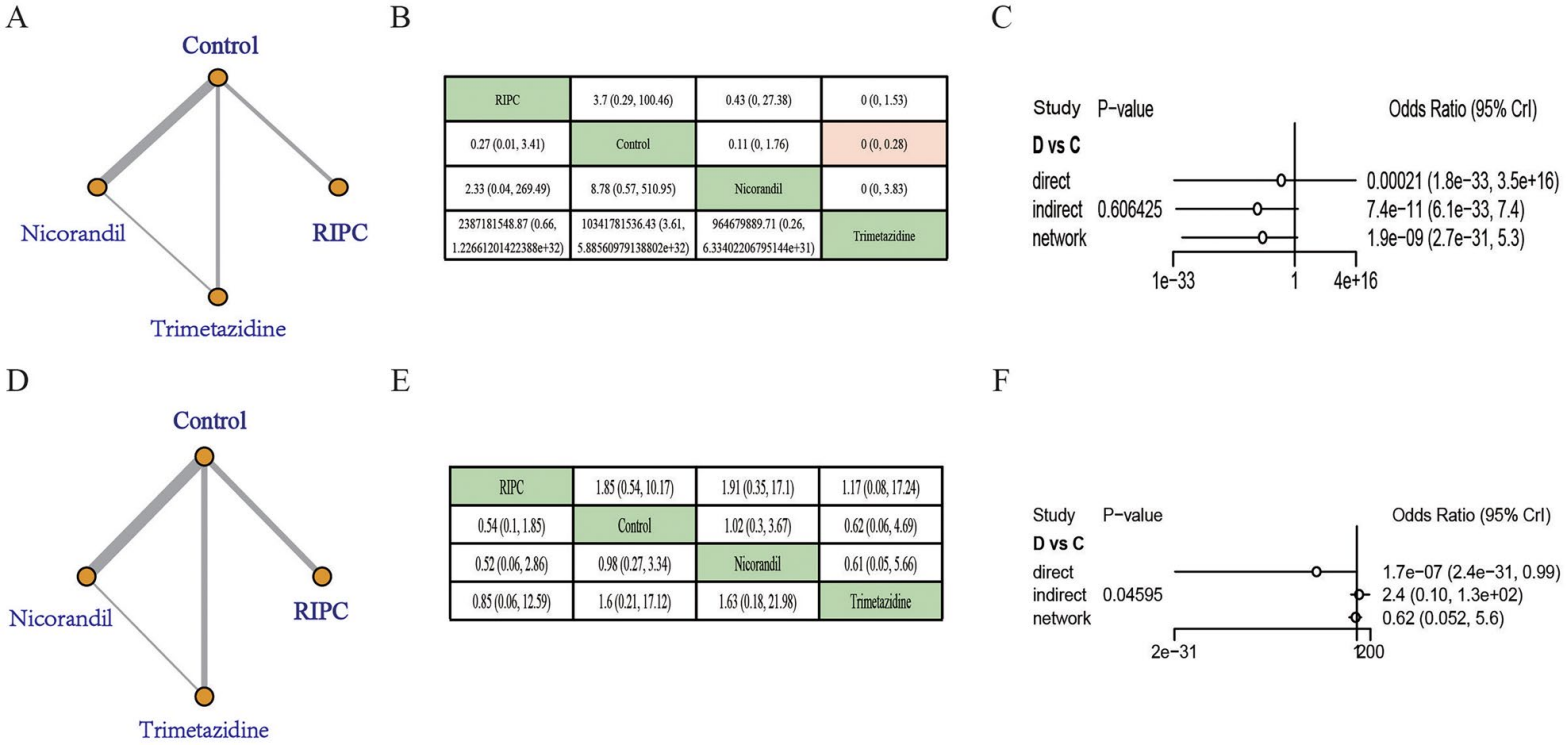
Effect of RIPC, nicorandil, and trimetazidine on additional outcomes including the requirement of hemodialysis and all-cause mortality. (C) Requirement of hemodialysis. (A) Network maps, (B) league tables, efficacy estimates with significant differences are in pink. (C) Consistency evaluation. (D–F) All-cause mortality. (D) Network maps, (E) league tables, and (F) consistency evaluation. A: RIPC; B: control; C: nicorandil; D: trimetazidine.

Eight studies reported the data on the requirement of hemodialysis [13,34,35,38–40,46,48]. Trimetazidine showed protective effects on the requirement of hemodialysis (Figure 5(A,B)). Consistency check results showed good consistency (*p* > .05) (Figure 5(C)). However, there was some heterogeneity in the included studies, and the comparison of RIPC versus controls presented a high degree of heterogeneity (Supplementary Table S8). In addition, there were no significant differences between RIPC, nicorandil, and the control groups in terms of all-cause mortality **(**Figure 5(D,E)**)**. Consistency check results showed inconsistency existed (*p* < .05) (Figure 5(F)). The heterogeneity of the analysis concerned with the impact of RIPC, nicorandil, and trimetazidine on all-cause mortality was low (Supplementary Table S9). Due to the lack of data on rehospitalization in the literature related to nicorandil and trimetazidine, we could not analyze the impact of these treatments on rehospitalization.

### 3.6. GRADE certainty of evidence

The protective effect of RIPC, nicorandil, and trimetazidine against CIN was proposed with moderate quality of evidence. Although all included studies were RCTs, there was some risk of bias in the studies due to some variation between the results, and the limited number of studies. Additionally, not all studies were placebo-controlled. These factors reduced the quality of this study from strong to moderate.

## 4. Discussion

A total of 40 RCTs were included in this NMA, which compared two potentially effective drugs (nicorandil and trimetazidine) commonly used in clinical practice and a noninvasive prophylactic measure (RIPC) based on hydration. This NMA aims to find more effective measures for the clinical prevention of CIN. Compared with a non-pharmacological preventive measure, it differed from the published meta-analysis. This study found that based on hydration, RIPC, nicorandil, and trimetazidine had prophylactic effects on CIN compared to control groups. However, there were no significant differences between the nicorandil, RIPC, and trimetazidine groups. Subgroup analysis results showed that there was still a protective effect in populations with mean eGFR <60 mL/min/1.73 m^2^ or with a high prevalence of diabetes mellitus.

It is well established that the primary pathogenesis of CIN is renal ischemia caused by an imbalance of various vasodilator and vasoconstrictor factors. Toxic injury to renal tubular epithelial cells, decreased nitric oxide production, intracellular calcium overload, and oxidative stress. In the process, oxygen free radicals may also cause renal tubular cell injury [52–56].

RIPC is a simple, feasible, and noninvasive intervention initially used to reduce ischemic injury in myocardial tissues. The standard of practice for RIPC is upper extremity RIPC. Typical steps in a RIPC implementation are three cycles of cuff inflation and deflation for 5 min, respectively [57]. Later, many studies have found that RIPC can also play a protective role in ischemic injuries in organs such as the kidneys and brain. Therefore, RIPC is considered to have a potential for clinical application [58]. Many published or ongoing RCTs have explored the protective effects of RIPC on organs during surgical procedures, including the heart [59], liver [60], kidney [61], and nervous system [62]. In addition, several studies have been reported to prevent CIN, with some of them finding a protective effect [5,63,64], while some did not [8,65]. In this study, there were 19 RCTs concerned with the impact of RIPC on the incidence of CIN, of which 10 suggested that RIPC had a preventive effect on CIN and nine indicated that it was ineffective. Resistance to ischemia–reperfusion injury after ischemic preconditioning stimuli is mediated by ischemia-associated ligand-activated cell-surface receptor ‘triggering mechanisms’ and by direct RISK pathway activation secondary to intracellular acidosis [66]. Trigger mechanism included PI3K/AKT/eNOS/GC/PKG, PKC, or JAK/STAT signal. The activation of low affinity A2b receptors for AKT and ERK 1/2 in the RISK pathway was strengthened by activated PKC. Then, the opening of the mitochondrial permeability transition pore (MPTP) was inhibited by glycogen synthase kinase-3-beta (GSK-3β), eventually protecting cells from apoptosis [67]. The NMA results showed that the RIPC has renal protective effects, which may be associated with this mechanism. However, further clinical and basic studies are needed for validation.

Nicorandil (2-nicotinamide ethyl nitrate) is a potassium (K) channel agonist, sensitive to adenosine triphosphate (ATP). In addition, it is also a nitric oxide donor. It has a diastolic effect on coronary vessels, especially small vessels, which in turn increases coronary blood flow and is commonly used in patients with angina pectoris [68]. It has been found that K-ATP channel activity attenuates injury by blocking reactive oxygen species (ROS) formation in a rat renal ischemia–reperfusion model [69]. It is well known that the kidneys are vascular-rich organs, including microvessels. It may be able to exert a similar protective effect in the kidneys as it does in the heart. Our study included 13 RCTs associated with the impact of nicorandil on the incidence of CIN, of which 11 suggested that nicorandil had a preventive impact on CIN, and two indicated that it was ineffective. The NMA results showed that the nicorandil also has renal protective effects. The following reasons may explain the results. First, nitrates improve nitric oxide production and increase the blood flow in the kidney. Meanwhile, nitrate reduces the production of intracellular oxygen free radicals and attenuates the inflammatory response [70,71]. In addition, opening K-ATP channels could reduce oxygen free radicals and expand the microvessels [72,73].

Trimetazidine, chemically known as 1-(2,3,4-trimethoxybenzyl) piperazine, is a commonly used anti-myocardial ischemic drug in clinical practice. It improves myocardial energy metabolism and maintains ATP levels, thus maintaining the normal action of the ion pump and stabilizing the intracellular environment. In addition, trimetazidine can reduce cellular damage by reducing oxygen free radicals, alleviating intracellular acidosis, and improving glucose metabolism [74,75]. There were eight RCTs on the effect of trimetazidine on the incidence of CIN included in this study, and all of them indicated that trimetazidine was effective in preventing or reducing the incidence of renal dysfunction caused by contrast media administration. Trimetazidine may exert its renoprotective effects through the following mechanisms. On the one hand, trimetazidine can prevent mitochondrial membrane dissipation, reduce the occurrence of Ca^2+^-induced mitochondrial swelling, and restore glutathione peroxidase levels [76]. On the other hand, trimetazidine increases ATP concentration by inhibiting nucleotide dephosphorylation, which in turn prevents ischemia [77]. Other studies have found that trimetazidine could decrease inflammatory cell infiltration [78], increase the expression of stathmin and hypoxia-inducible factor 1 alpha, then promote renal tubular repair [79,80].

Otsuka et al. [36] compared RIPC and nicorandil on the basis of hydration therapy only and found that the incidence of early elevation of SCr in patients who received RIPC was significantly lower than the control groups, while no significant difference between the nicorandil and control groups. Another study compared nicorandil and trimetazidine based on hydration therapy with hydration only and suggested that preoperative administration of regular doses of trimetazidine or intravenous nicorandil may reduce the incidence of CIN, and combining the two drugs may provide further benefit [46].

The SUCRA results showed that nicorandil ranked 1st, trimetazidine ranked 2nd, and RIPC ranked 3rd, all higher than controls in preventing CIN. However, there were no significant differences between the nicorandil, RIPC, and trimetazidine groups, and this NMA showed that combining RIPC, nicorandil, or trimetazidine with hydration may be more effective in preventing CIN. RIPC is a simple, feasible, and noninvasive intervention with no obvious side effects; RIPC may be the better intervention than these two drugs in preventing CIN. To date, no RCTs of RIPC administered with nicorandil or trimetazidine were searched. Whether RIPC in combination with nicorandil or trimetazidine would be more effective than one intervention alone needs to be further explored by designing large sample RCTs. Trimetazidine showed protective effects on the requirement of hemodialysis. Since the small number of studies on trimetazidine, a larger sample of RCT studies is needed to analyze further. In addition, these three therapeutic measures did not significantly reduce all-cause mortality rates. Patients in our studies were suffering from heart disease. The impact of heart disease on mortality needs to be considered. There were 11 studies with mortality outcomes. We assessed bias due to cardiac problems by evaluating whether the two groups differed between cardiac-related histories, such as infarction arrhythmia, and cardiac-related indices, such as LVEF or BNP. The cardiac-related histories and markers of the intervention and control groups were not different at baseline. Seven of these studies reported both all-cause and cardiac mortality, and neither all-cause nor cardiac mortality differed between the intervention and control groups. Therefore, we thought that the effect of heart problems in our study was not noticeable. However, consistency check results showed inconsistency existed (*p* < .05). The reason for inconsistency may be the small number of studies.

There are some limitations in this NMA. First, this study only analyzed the effect of two pharmacological interventions and RIPC on preventing CIN, and the conclusion could not be generalized to other drugs. Second, the number of included RCTs was limited. This NMA only included 40 RCTs, and the presence of chance cannot be denied. Further studies can analyze additional treatments and include more studies for comprehensive analysis. Third, there was some heterogeneity in the included studies, such as the surgery patients accepted. Fourth, hydration therapy was used both in the intervention and control groups. No other drugs reported to have a preventive effect on CIN were used. However, some studies did not mention whether a placebo was used in control groups. This may lead to some bias in the results of the study. Fifth, the results of all-cause mortality and the requirement of hemodialysis need to be analyzed further due to the limited number of studies that have included these data.

## 5. Conclusions

Nicorandil, trimetazidine, and RIPC all showed renal protective effects. Based on hydration, nicorandil, trimetazidine, and RIPC may show better prophylaxis against CIN than hydration alone after intravenous contrast administration. However, further confirmation is needed from clinical studies with larger samples.

## Supplementary Material

Appendix A Supplementary_materials new.doc

## Data Availability

The data underlying this article are available in the article and in its online supplementary material.
