# Supplementary material for: Effect of remote ischemic preconditioning, nicorandil, and trimetazidine in contrast-induced nephropathy: a network meta-analysis of randomized controlled trials
Source: Ren Fail. 2024 Nov 27;46(2):2431141. doi: 10.1080/0886022X.2024.2431141 (PMC11610246; doi:10.1080/0886022X.2024.2431141)
Supplement: Appendix A Supplementary_materials new.doc [file IRNF_A_2431141_SM8021.doc]

**Caption for supplementary materials**

**Search strategy 2**

**PICOS outline 4**

**Characteristics and baseline information of the enrolled publications 5**

**Quality assessment 15**

**Harbord test 19**

**Sensitivity analysis 19**

**Analysis of heterogeneity 20**

**Trajectory density graphs 23**

[**The SUCRA results 28**](#__RefHeading___Toc5964)

[**The GRADE results 29**](#__RefHeading___Toc5964)

1. **Search strategy**

| **Detailed search strategy of PubMed** | |
| --- | --- |
| **Search** | **Query** |
| #1 | "Ischemic Preconditioning" [Mesh] |
| #2 | (preconditioning, ischemic) OR (ischemic pre-conditioning) OR (ischemic pre conditioning) OR (pre-conditioning, ischemic) OR (ischaemic preconditioning) OR (ischemia preconditioning) OR (remote ischemic preconditioning) OR (remote ischaemic preconditioning) OR (remote ischemia preconditioning) OR (RIPC) OR (preconditioning, ischemia) OR (ischemia pre-conditioning) OR (ischemia pre conditioning) OR (pre-conditioning, ischemia) OR (preconditioning, ischaemic) OR (ischaemic pre-conditioning) OR (ischaemic pre conditioning) OR (pre-conditioning, ischaemic) |
| #3 | #1 OR #2 |
| #4 | "Nicorandil"[Mesh] |
| #5 | (2-Nicotinamidoethyl Nitrate) OR (2 Nicotinamidoethyl Nitrate) OR (Nitrate, 2-Nicotinamidoethyl) OR (2-Nicotinamidethyl Nitrate) OR (2 Nicotinamidethyl Nitrate) OR (Nitrate, 2-Nicotinamidethyl) OR (SG-75) OR (SG 75) OR (SG75) OR (Ikorel) OR (Adancor) OR (Dancor) OR ((2 hydroxyethyl) nicotinamide nitrate) OR (2 (nicotinamido) ethyl nitrate) OR (2 nicotinoamidoethyl nitrate) OR (angicor) OR (n (2 hydroxyethyl) nicotinamide nitrate) OR (n (2 nitratoethyl) nicotinamide) OR (n [2 (nitrooxy) ethyl] 3 pyridinecarboxamide) OR (perisasol) OR (rp 46417) OR (rp46417) OR (sigmart) |
| #6 | #4 OR #5 |
| #7 | "Trimetazidine"[Mesh] |
| #8 | (Centrophène) OR (Trimetazidine Dihydrochloride) OR (Dihydrochloride, Trimetazidine) OR (Vastarel) OR (Trimétazidine Irex) OR (Vasartel) OR (Idaptan) |
| #9 | #7 OR #8 |
| #10 | #3 OR #6 OR #9 |
| #11 | "Contrast Media" [Mesh] |
| #12 | (Contrast) OR (Media, Contrast) OR (Contrast Agent) OR (Agent, Contrast) OR (Contrast Materials) OR (Materials, Contrast) OR (Contrast Agents) OR (Agents, Contrast) OR (Contrast Material) OR (Material, Contrast) OR (Radiocontrast Media) OR (Media, Radiocontrast) OR (Radiocontrast Agent) OR (Agent, Radiocontrast) OR (Radiocontrast Agents) OR (Agents, Radiocontrast) OR (Radiopaque Media) OR (Media, Radiopaque) OR (contrast dye) OR (radiocontrast medium) OR (radiography contrast medium) OR (roentgen contrast medium) OR (contrast medium) |
| #13 | "Angiography" [Mesh] |
| #14 | (angiographical method) OR (angioradiology) OR (blood vessel radiography) OR (moving table angiography) OR (peripheral angiography) OR (peripheral vasculography) OR (rheoacroangiography) OR (Angiographies) OR (Angiogram) OR (Angiograms) OR (Arteriography) OR (Arteriographies) |
| #15 | #11 OR #12 OR #13 OR #14 |
| #16 | "Randomized Controlled Trials as Topic"[Mesh] |
| #17 | (Randomized Controlled Trials) OR (Controlled Clinical Trials) OR (Clinical Trials, Randomized) OR (Trials, Randomized Clinical) OR (Controlled Clinical Trials, Randomized) OR (randomized) OR (Randomly) OR (randomization) OR (pragmatic clinical trials as topic) OR (randomized controlled trials as topic) OR (randomized controlled trial (topic)) |
| #18 | #16 OR #17 |
| #19 | #10 AND #15 AND #18 |

| **Detailed search strategy of Embase** | |
| --- | --- |
| **Search** | **Query** |
| #1 | 'Ischemic Preconditioning'/exp |
| #2 | 'ischaemic preconditioning' OR 'ischemic preconditioning' OR 'ripc' OR 'preconditioning, ischemia' OR 'remote ischemic preconditioning'/exp OR 'remote ischemic preconditioning' OR 'remote ischaemic preconditioning'/exp OR 'remote ischaemic preconditioning' OR 'remote ischemia preconditioning' OR 'ischemia preconditioning' OR 'pre-conditioning, ischemic' OR 'ischemic pre-conditioning' OR 'ischemic pre conditioning' OR 'preconditioning, ischemic' OR 'ischemia pre-conditioning' OR 'ischemia pre conditioning' OR 'pre-conditioning, ischemia' OR 'preconditioning, ischaemic' OR 'ischaemic pre-conditioning' |
| #3 | #1 OR #2 |
| #4 | "Nicorandil"[Mesh] |
| #5 | '2-Nicotinamidoethyl Nitrate' OR '2 Nicotinamidoethyl Nitrate' OR 'Nitrate, 2-Nicotinamidoethyl' OR '2-Nicotinamidethyl Nitrate' OR '2 Nicotinamidethyl Nitrate' OR 'Nitrate, 2-Nicotinamidethyl' OR 'SG-75' OR 'SG 75' OR 'SG75' OR 'Ikorel' OR 'Adancor' OR 'Dancor' OR '(2 hydroxyethyl) nicotinamide nitrate' OR '2 (nicotinamido) ethyl nitrate' OR '2 nicotinoamidoethyl nitrate' OR 'angicor' OR 'n (2 hydroxyethyl) nicotinamide nitrate' OR 'n (2 nitratoethyl) nicotinamide' OR 'n [2 (nitrooxy) ethyl] 3 pyridinecarboxamide' OR 'perisasol' OR 'rp 46417' OR 'rp46417' OR 'sigmart' |
| #6 | #4 OR #5 |
| #7 | 'trimetazidine'/exp |
| #8 | ‘Centrophène' OR 'Trimetazidine Dihydrochloride' OR 'Dihydrochloride, Trimetazidine' OR 'Vastarel' OR 'Trimétazidine Irex' OR 'Vasartel' OR 'Idaptan’ |
| #9 | #7 OR #8 |
| #10 | #3 OR #6 OR #9 |
| #11 | 'contrast medium'/exp |
| #12 | 'contrast media' OR 'Contrast' OR 'Media, Contrast' OR 'Contrast Agent' OR 'Agent, Contrast' OR 'Contrast Materials' OR 'Materials, Contrast' OR 'Contrast Agents' OR 'Agents, Contrast' OR 'Contrast Material' OR 'Material, Contrast' OR 'Radiocontrast Media' OR 'Media, Radiocontrast' OR 'Radiocontrast Agent' OR 'Agent, Radiocontrast' OR 'Radiocontrast Agents' OR 'Agents, Radiocontrast' OR 'Radiopaque Media' OR 'Media, Radiopaque' OR 'contrast dye' OR 'radiocontrast medium' OR 'radiography contrast medium' OR 'roentgen contrast medium' |
| #13 | 'angiography'/exp |
| #14 | 'angiographical method' OR 'angioradiology' OR 'blood vessel radiography' OR 'moving table angiography' OR 'peripheral angiography' OR 'peripheral vasculography' OR 'rheoacroangiography' OR 'angiography' OR 'angiographies' OR 'angiogram' OR 'angiograms' OR 'arteriography' OR 'arteriographies' |
| #15 | #11 OR #12 OR #13 OR #14 |
| #16 | 'randomized controlled trial (topic)'/exp |
| #17 | 'Randomized Controlled Trials' OR 'Controlled Clinical Trials' OR 'Clinical Trials, Randomized' OR 'Trials, Randomized Clinical' OR 'Controlled Clinical Trials, Randomized' OR 'randomized' OR 'Randomly' OR 'randomization' OR 'pragmatic clinical trials as topic' OR 'randomized controlled trials as topic' OR 'randomized controlled trial (topic)' |
| #18 | #16 OR #17 |
| #19 | #10 AND #15 AND #18 |

1. **PICOS outline**

| **PICOS outline** |  |
| --- | --- |
| Population | Patients undergoing an interventional or diagnostic radiological procedure which needs to use contrast media. |
| Intervention | Remote ischemic preconditioning, nicorandil and trimetazidine. |
| Comparison | Placebo |
| Outcome | 1) Main outcomes: Overall ranking of all the interventions on the incidence of contrast-induced nephropathy.  2) Subgroup analysis of the incidence of contrast-induced nephropathy in patients at different risk or with different drug dose.  3) Additional outcomes: the effect of the interventions on the incidence of hospitalization, hemodialysis, and mortality. |
| Study design | Randomized-controlled trial. |

1. **Characteristics and baseline information of the enrolled publications**

**Table S1: Characteristics and baseline information of the enrolled publications**

|  | Baseline characteristics of the subjects | | | | |  |  | Characteristics of the trials | | | | | |  |
| --- | --- | --- | --- | --- | --- | --- | --- | --- | --- | --- | --- | --- | --- | --- |
| Study | years old  (mean ± SD/SE/range) | Sex distribution  (Male sex, (%) | Diabetes distribution  (%) | Hypertention distribution  (%) | 基础 eGFR  [ml/(min·1.73m^2^)] [mean ± SD/ median (IQR)] | |  | contrast medium  Dose (mL) | Surgery  (operation procedure) | Duration | Baseline medication | Interventions, dosage  (N) | Controls (N) | outcomes |
| Guo 2019 | RIPC:  71.69 ± 6.69  Control:  70.81 ± 6.27 | RIPC: 61.8  Control: 56.4 | RIPC:  38.2  Control: 41.8 | NA | RIPC:  80.95 ± 25.38  Control:  79.53 ± 27.00 | |  | RIPC:  141 ± 59.5  Control:  143 ± 58.7 | PCI | 1 month | Hydration | RIPC: 3 cycles, 2 hours to surgery (110) | Placebo  (110) | Incidence of CIN; the changes in creatinine from baseline to 48 h and changes in NGAL and KIM-1 from baseline to 12 and 48 h after contrast exposure |
| Castillo 2022 | RIPC:  73.85 ± 6.9  Control:  72.91 ± 5.7 | RIPC: 50  Control: 48.3 | RIPC:  13.3  Control: 15.8 | RIPC: 31.7  Control: 36.7 | RIPC:  65.34 ± 22.0  Control:  64.67 ± 19.9 | |  | NA | Elective aortic surgery treated by endovascular repair | 1 month | Hydration | RIPC: 4 cycles, 12 hours to surgery(60) | NA  (60) | Incidence of CIN; hematocrit levels and urinary albumin: creatinine ratio at 48 hr and 30 days |
| Yan 2021 | RIPC:  65 ± 10  Control:  65 ± 12 | RIPC: 77.5  Control: 73.6 | RIPC:  21.1  Control: 14.3 | RIPC: 56.8  Control: 56.1 | RIPC: 66 ± 21  Control: 65 ± 20 | |  | RIPC: 23 ± 58  Control: 217 ± 61 | PCI | 72 hours | Hydration | RIPC: 3 cycles, 30 minutes to surgery(142) | Placebo  (140) | Incidence of CIN; the changes in creatinine CysC and eGFR from baseline to 24 h, 72h after contrast exposure |
| Otsuka 2021 | RIPC:  70 ± 10  Nicorandil:  70 ± 8  control:  72 ± 9 | RIPC:  86  Nicorandil: 84  control:  71 | RIPC:  46  Nicorandil: 49  control:  50 | RIPC:  86  Nicorandil: 83  Control:  88 | RIPC: 63 ± 15  Nicorandil: 64 ± 19  control: 64 ± 17 | |  | RIPC:  105(80-121)  Nicorandil: 102(70-130)  control:  100(80-130) | PCI | 24 hours | Hydration | RIPC: 3 cycles, 1 hour to surgery(69)  Nicorandil: 4 mg of nicorandil was intraven-  ously administered for 5 min at least 1 h before PCI, followed by continuous infusion of nicorandil (6 mg/h) for at least 8 h during PCI (77) | NAT  (74) | Incidence of the early increase in SCr after PCI |
| Moradkhani 2020 | RIPC:  56.3 ± 11.8  Control:  56.8 ± 11.4 | RIPC: 58.6  Control: 54.3 | RIPC:  18.6  Control: 24.3 | NA | RIPC:  79.25 ± 16.38  Control:  77.7 ± 15.29 | |  | NA | CA | 48 hours | Hydration | RIPC: 3 cycles, 1 hour to surgery (70) | Placebo  (70) | Acute renal failure development; the serum creatinine, eGFR, and serum CysC changes compared to the baseline |
| Sampathkumar 2019 | RIPC:  61.0 ± 8.6  Control:  58.6 ± 7.3 | RIPC:  72  Control: 82 | RIPC: 70  Control: 68 | RIPC: 54  Control: 44 | RIPC:  54.95 ± 10.0  Control:  54.8 ± 9.65 | |  | RIPC:  123 ± 53  Control:  127.5 ± 61.0 | CA | 1 month | Hydration | RIPC: 4 cycles, 1 hour to surgery (50) | Placebo  (50) | Incidence of CIN; the change in serum creatinine from day 0 to day 2 after contrast administra-  tion, duration of hospital stay, requirement of hemodialysis, rehospitalisation, and one-month mortality |
| Roubille1 2019 | RIPC:  75.6 ± 9.2  Control:  76.7 ± 7.6 | RIPC:  69  Control: 69 | RIPC: 55  Control: 51 | RIPC: 89  Control: 88 | RIPC: 43 ± 11  Control: 42 ± 10 | |  | RIPC:  80(50-111)  Control:  73 (45-121) | CA/ PTCA | 1 year | Hydration | RIPC: 4 cycles, from 5 minutes to 1 hour to surgery (98) | Placebo  (104) | Incidence of CIN; serum creatinine and eGFR levels from baseline up to 48hours and 12 months after contrast medium administration, the incidence of major clinical events at 12 months |
| Zhou 2018 | RIPC:  69.42 ± 7.07  Control:  69.14 ± 7.80 | RIPC: 60.0  Control: 61.4 | RIPC: 48.00  Control: 47.37 | RIPC:  56.00  Control: 56.14 | RIPC:  69.994 ± 8.908  Control:  68.023 ± 10.168 | |  | RIPC:  114.76 ± 44.22  Control:  108.82 ± 43.25 | PCI | 6 months | Hydration | RIPC: 4 cycles, 1 hour to surgery (50) | NAT  (57) | Incidence of CIN; the changes in Scr, Cysc, eGFR, NGAL levels before and after PCI;renal failure undergoing dialysis or kidney transplantation, readmission, and death |
| Wojciechowska 2018 | RIPC:  64.4 ± 10.5  Control:  62.9 ± 8.6 | RIPC:  67.7  Control: 68.9 | RIPC:  21.0  Control: 46.2 | RIPC: 72.6  Control: 77.1 | RIPC:  114.76 ± 44.22  Control:  108.82 ± 43.25 | |  | RIPC:  155.5 ± 76.7  Control:  143.7 ± 55.7 | PCI | 1 year | Hydration | RIPC: 3 cycles,＜2 hour to surgery(62) | Placebo  (61) | Incidence of CIN; MACCE |
| Elserafy 2018 | RIPC:  65.16 ± 7.98  Control:  65.1 ± 7.07 | RIPC:  58  Control: 58 | RIPC: 66  Control: 66 | RIPC: 64  Control: 68 | RIPC:  45.81 ± 7.89  Control:  44.60 ± 7.72 | |  | RIPC:  125.20 ± 37.59  Control:  119.80 ± 38.41 | PCI | 48 hours | Hydration | RIPC: 4 cycles, ＜45 minutes to surgery(50) | Placebo  (50) | Incidence of CIN |
| Shamilevich 2017 | RIPC:  60.5 ± 1.95  Control:  62.96 ± 1.72 | RIPC: 80.8  Control:  88.0 | RIPC: 30.8  Control: 32 | NA | RIPC:  62.24 ± 5.3  Control:  71.88 ± 6.1 | |  | RIPC:  155.8 ± 16.9  Control:  148.3 ± 16.7 | CA | 60 hours | Hydration | RIPC: 3 cycles, 1 hour to surgery (26) | Placebo  (25) | Incidence of CIN; death, myo-  cardial infarction, acute coronary syndrome and need for hemodia-  lysis during index hospitalization |
| Kahlert 2017 | NA | NA | NA | NA | NA | |  | NA | TF-TAVI | 1 year | Hydration | RIPC: 3 cycles, 30 minutes to surgery (50) | Placebo  (50) | Myocardial injury; the inciden-  ces of periprocedural myocardial infarction, delayed gadolinium enhancement on postprocedural cardiac MRI, AKI, periprocedu-  ral stroke, and the incidence and volume of new lesions on postprocedural cerebral MRI |
| Singh 2016 | RIPC:  67.8 ± 7.6  Control:  69.0 ± 8.6 | RIPC: 45.1  Control: 49 | RIPC:  100  Control: 100 | RIPC: 80.4  Control: 90.2 | RIPC:  48.5 ± 12.0  Control:  46.6 ± 10.2 | |  | RIPC:  197.5 ± 114.3  Control:  196.4 ± 118.8 | PCI | 72 hours | Hydration | RIPC: 3 cycles, 30 minutes to surgery (51) | Placebo  (51) | Incidence of CIN;the changes in creatinine, NGAL level, cTnT, CKMB and hs-CRP over time from baseline |
| Menting 2015 | RIPC:  71 ± 11  Control:  73 ± 8.5 | RIPC:  39  Control: 58 | RIPC: 22  Control:28 | RIPC: 78  Control: 67 | RIPC:  51 ± 11  Control:  52 ± 13 | |  | RIPC: 99 ± 29  Control: 98 ± 29 | Interventional/ diagnostic radiological procedure | 6 weeks | Hydration | RIPC: 4 cycles, ＜45 minutes to surgery (36) | Placebo  (36) | The change in serum creatinine from baseline to 48-72 hours after contrast administration; incidence of CIN; rehospitaliza-  tion, hemodialysis, and mortality |
| Healy 2015 | RIPC:  63 (57- 69)  Control:  62 (57- 67) | RIPC:  51  Control: 59 | NA | RIPC: 41  Control:44 | NA | |  | NA | CECT-scan | 48 hours | Hydration | RIPC: 3 cycles, 40 minutes to surgery (43) | NAT  (44) | The change in SCr after the CECT-scan; serum urea at 24 and 48 hours after the CTscan, incidence of reduced urine output within 48 hours of the scan, length of hospital stay |
| Gholoobi 2015 | RIPC:  67.08 ± 12.49  Control:  70.31 ± 11.18 | RIPC:  49  Control: 51 | RIPC:  76  Control: 69.2 | RIPC: 75  Control: 76 | NA | |  | NA | CA/ coronary angioplasty | 48 hours | Hydration | RIPC: 4 cycles, 1 hour to surgery (25) | Placebo  (26) | The changes in serum creatinine, eGFR, CIN incidence, Mehran score, and the volume of saline infused within the 48 hours following the angiography |
| Igarashi 2013 | RIPC: 　　 71.3 ± 8.1  Control:  70.8 ± 7.6 | RIPC: 66.7  Control: 76.7 | NA | NA | RIPC: 47.4 ± 9.4  Control: 48.9 ± 6.0 | |  | RIPC:  92.9 ± 33.2  Control:  91.8 ± 39.4 | CA/ PCI | 48 hours | Hydration | RIPC: 4 cycles, 2 hour to surgery (30) | NA  (30) | Incidence of CIN; the level of hs-CRP, PTX3, the derivatives of reactive oxidative metabolites (asymmetrical dimethylarginine (ADMA) at 24h, and serum  creatinine, eGFR and cystatin C at 48h after contrast medium  exposure |
| Uzun 2022 | RIPC:  59.7 ± 8.8  Control:  59.4 ± 9.8 | RIPC: 55.1  Control: 60 | RIPC: 33.9  Control: 24 | RIPC: 64.4  Control: 62 | RIPC: 95 ± 22  Control: 93±28 | |  | RIPC: 86 ± 43  Control: 76 ± 36 | CA | 72 hours | Hydration | RIPC: 3 cycles, 10-45 minutes to surgery (118) | Placebo  (50) | Rate of prevention of CIN between lower limb and upper limb RIPC; Δcreatinine levels, duration of hospitalization, my-  ocardial infarction status during the procedure and on mortality during hospitalization |
| Shafipoor 2021 | RIPC: 66.87±10.57 Control:  67.94 ± 10.28 | RIPC: 67.4  Control: 68.2 | RIPC:  40.7  Control: 78.8 | RIPC: 64.4  Control: 62 | RIPC:  47.13 ± 15.94  Control:  48.38 ± 10.32 | |  | NA | CA/coronary angioplasty | 48 hours | Hydration | RIPC: 4 cycles, 45 minutes to surgery (86) | Placebo  (85) | Incidence of CIN |
| Nawa 2015 | Nicorandil: 70.4 ± 7.7  control:  70.1 ± 8.1 | Nicorandil: 81.6  control: 78.7 | Nicorandil: 58 .2  control:  50 | Nicorandil: 70.4  control:  72.3 | Nicorandil:  59.6 ± 16.5  control:  58.1 ± 16.4 | |  | Nicorandil:  135.2 ± 57.0 control:  146.3 ± 63.6 | PCI | 1 month | Hydration | Nicorandil:  2 vials of nicorandil (48 mg/V) dissolve in 100 mL 0.9% saline, and dripped it at speed of 0.1 mL/kg/h) plus 0.9% saline hydration intrave-  nously infused at 1.0 mL/kg/h(106) | Placebo  (107) | Incidence of CIN; percent rise in serum creatinine and cystatin C, percent decline in eGFR within 1 month after the procedure |
| Ko 2013 | Nicorandil: 70.8 ± 9.6  control:  69.1 ± 10.3 | Nicorandil: 72.6  control: 67.1 | Nicorandil: 41.1  control: 55.3 | Nicorandil: 78.1  control:  80.3 | Nicorandil:  37.5 ± 13.4  control:  40.1 ± 13.9 | |  | Nicorandil:  125.6 ± 69.1  control: 126.9±74.6 | CA | 48 hours | Hydration | Nicorandil:  12 mg nicorandil was diluted in 100 mL of 0.9% saline and adminis-  tered intravenously over a 30-minute period just prior to coronary angio-  graphy  (73) | Placebo  (76) | Incidence of CIN; maximal increase in serum Cr and cystatin C levels within 48 hours after the procedure. MAEs including death, myocardial infarction, stroke, renal failure requiring dialysis, and acute pulmonary edema |
| Yusuf 2024 | Nicorandil: 60.33 ± 8.731  control:  60.46 ± 8.175 | Nicorandil: 87.6  control: 82.9 | Nicorandil: 35.2  control:  39 | Nicorandil: 28.6  control:  41.9 | Nicorandil:  63.64 ± 9.088  control:  60.26 ± 10.781 | |  | Nicorandil: 118.57 ± 49.738  control:  128.00 ± 55.598 | PCI | 72 hours | Hydration | Nicorandil:  oral nicorandil (10 mg, 3 times/d) 1 day before procedure and for 2 days after PCI (105) | NAT  (105) | Incidence of CIN; MAEs (including all-cause mortality, cardiac death, myocardial infarction, acute heart failure, end-stage kidney disease and minor or major bleed) |
| Moghaddam 2023 | Nicorandil: 60.97 ± 9.68  control:  60.61 ± 10.17 | Nicorandil: 45.34  control: 41.86 | Nicorandil: 57.55  control: 62.21 | Nicorandil: 66.86  control: 64.53 | Nicorandil:  78.91 ± 28.65  control:  83.54 ± 28.81 | |  | Nicorandil:  41.97 ± 12.98  control:  43.52 ± 14.18 | Cardiac catheterizati-  on | 48 hours | Hydration | Nicorandil:  oral Nicorandil (10 mg daily, three times) from 2 h before to 48 h after the catheterization (172) | NA  (172) | Incidence of CIN; Kidney function before and after the injection of contrast agent |
| Zhang 2020 | Nicorandil: 67.25 ± 6.42  control:  67.11 ± 7.19 | Nicorandil: 78.7  control: 76 | Nicorandil: 22.7  control: 23.3 | Nicorandil: 46  control:  47.3 | Nicorandil:  124.00 ± 15.16  control:  123.54 ± 14.37 | |  | Nicorandil: 145.45 ± 10.68  control:  144.50 ± 10.56 | PCI | 14 days | Hydration | Nicorandil:  oral Nicorandil (10 mg three times daily) starting 1 day before, and ending 3 days after PCI (150) | NAT  (150) | Incidence of CIN; changes in BUN, Scr, Ccr,and Cys-C within 72 hours after PCI, and post-PCI major adverse clinical events |
| Zhang 2020-1 | Nicorandil: 67.4 ± 6.6  control:  67.0 ± 7.2 | Nicorandil: 74.4  control: 71.2 | Nicorandil: 19.2  control: 23.2 | NA | Nicorandil:  51.2 ± 4.1  control:  51.0 ± 3.8 | |  | Nicorandil:  166.4 ± 49.6  control:  167.0 ± 46.7 | PCI | 72 hours | Hydration | Nicorandil:  nicorandil take orally  (10 mg, 3 times/d) 1 day before operation and for 3 days after PCI (125) | NAT  (125) | Incidence of CIN; the changes of BUN, Scr, and crCl within 72 hours and MAEs that occurred during hospitalization, including all-cause mortality, hypotension or severe drop in blood pressure, renal replacement therapy, internal bleeding, emergency PCI or surgical coronary bypass, acute heart failure after PCI, and cerebrovascular events |
| Zeng 2019 | Nicorandil Usual-dose group:  67.09 ± 6.85  Nicorandil Double-dose group:  65.37 ± 7.19  control:  66.69 ± 7.33 | Nicorandil Usual-dose group:  68.2  Nicorandil Double-dose group:  70.2  control:  39.8 | Nicorandil Usual-dose group:  19.6  Nicorandil Double-dose group:  17.1  control: 16.1 | Nicorandil: Usual-dose group: 64.5  Nicorandil Double-dose group: 37.8  control: 52.7 | Nicorandil: Usual-dose group:  77.41 ± 16.82  Nicorandil Double-dose group:  80.20 ± 23.98  control:  81.42 ± 26.84 | |  | Nicorandil: Usual-dose group:  172.83 ± 49.66  Nicorandil Double-dose group:  184.79 ± 53.05  control:  184.79 ± 53.05 | CA/ PCI | 48 hours | Hydration | Nicorandil:  The double-dose group:  (n=111, 30 mg/day)  the usual-dose group:  (n=107, 15 mg/day),  diluted in 100 mL of 0.9% saline three times daily (beginning 2 days prior to the coronary  intervention and continu-  ing 2 days after it | Placebo  (112) | Incidence of CIN; the changes in BUN, Cys-C, SCr, eGFR, and CRP within 48h after contrast agent exposure and MAEs (including all-cause mortality, renal replacement therapy, stroke, acute heart failure, and pulmonary edema) occurring, during hospitalization and 14-day follow-up period |

| Fan 2019 | Nicorandil: 62.25 ± 16.63  control:  65.87 ± 17.62 | Nicorandi: 59.84  control: 53.6 | Nicorandil: 63.78  control:  60 | Nicorandil: 53.54  control:  49.6 | Nicorandil:  59.32 ± 19.31  control:  61.75 ± 22.56 |  | Nicorandil:  128.39 ± 37.25  control:  122.81 ± 35.92 | PCI | 12.8 months | Hydration | Nicorandil:  10 mg of nicorandil three times per day, from 2 days before to 2 days after an elective coronary procedure (127) | NA  (125) | Incidence of CIN; all-cause mortality, stroke, non-fatal myo-  cardial infarction, percutaneous coronary revascularization, coro-  nary artery bypass graft surgery, congestive heart failure, pulmo-  nary edema, and end-stage renal disease |
| --- | --- | --- | --- | --- | --- | --- | --- | --- | --- | --- | --- | --- | --- |
| Zhao 2018 | Standard nicorandil:  63.26 ± 4.91  Intensive nicorandi:  65.27 ± 6.12  control:  64.71 ± 5.68 | Standard nicorandil:  57.14  Intensive nicorandi:  52  control: 52.94 | Standard nicorandil:  100  Intensive nicorandi:  100  control: 100 | Standard nicorandil:  64.71  Intensive nicorandi:  63.27  control: 68 | Standard nicorandil:  120.1 ± 5.86  Intensive nicorandi:  121.3 ± 6.2  control:  119.6 ± 5.5 |  | Standard nicorandil: 245.87 ± 48.69  Intensive nicorandi:  250.87 ± 50.72  control:  241.85 ± 49.71 | CA | 3 days | Hydration | standard nicorandil  group:  (n=49, 5 mg, 3 times/d)  intensive nicorandil  group:  (n=50, 3 times/d)  2 days before and 3 days after angiography | NA  (51) | Incidence of CIN; 25% or greater  reduction in the eGFR compared to baseline, which was calculated by using MDRD equation and Scr obtained before PCI and within 3 days after PCI |
| Iranirad 2017 | Nicorandil: 61.35 ± 11.77  control:  57.64 ± 12.42 | Nicorandil: 60.9  control: 62.5 | Nicorandil: 42.2  control: 40.6 | Nicorandil: 54.7  control: 64.1 | Nicorandil:  76.39 ± 24.6  control:  83 ± 28.1 |  | Nicorandil: 213.98 ± 44.6  control:  202.26 ± 44.4 | PCI | 72 hours | Hydration | Nicorandil:  10 mg nicorandil, daily, from 30 min before to 3 days after the procedure (64) | NA  (64) | Incidence of CIN; the changes in SCr and eGFR within 72 h after contrast agent exposure |
| Fan 2016 | Nicorandil: 66.07 ± 6.37  control:  67.37 ± 6.33 | Nicorandi: 73.33  control: 79.17 | Nicorandil: 55  control: 51.67 | Nicorandil: 57.5  control: 61.67 | Nicorandil:  49.62 ± 5.38  control:  50.38 ± 5.74 |  | Nicorandil:  145.3 ± 51.6  control:  149.2 ± 57.0 | CA | 72 hours | Hydration | Nicorandil:  10 mg nicorandil three times daily from 2 days before to 3 days after contrast media exposure (120) | Placebo  (120) | Incidence of CIN; the changes of SCr, Cys-C and eGFR within 72 h after the procedure; and (2) major adverse events occurring within 30 days after contrast exposure, including all-cause death, renal failure requiring dialysis, myocardial infarction,  stroke, and worsening heart failure defined as a deteriorated  NYHA functional class were recorded |

| Cheng 2018 | NA | Nicorandi: 74.3  Trimetazidine:  72.1  Nicorandil+  Trimetazidine:  73.1  control:  74 | Nicorandil: 61.9  Trimetazidine:  64.4  Nicorandil+  Trimetazidine:  65.4  control: 63.5 | NA | Nicorandil:  64.6 ± 15.2  Trimetazidine:  65.1 ± 16.3  Nicorandil+  Trimetazidine:  67.6±11.5  control:  65.2 ± 13.1 |  | Nicorandil: 166.81 ± 29.21  Trimetazidine:  159.27 ± 31.73  Nicorandil+  Trimetazidine:  157.92±30.34  control:  164.99 ± 34.27 | PCI | 72 hours | Hydration | Nicorandil:  Dissolve 84 mg of nicorandil (12 mg/ couple) in 100 ml of saline at a rate of 0.1 ml/ (kg-h) starting 6 h before surgery and until 12 h after the operation (n=105)  Trimetazidine group: 20 mg, 3 times/d orally from 2 d preoperatively to 3 d postoperatively (n=104)  Nicorandil+  Trimetazidine:  On top of the medication in the nicorandil group.  Trimetazidine (20 mg, 3 times/d) was administered orally from 2 d preoperatively to 3 d postoperatively (n=104) | Placebo  (208) | Incidence of CIN; all-cause death, renal failure requiring dialysis; stroke, acute heart failure |
| --- | --- | --- | --- | --- | --- | --- | --- | --- | --- | --- | --- | --- | --- |
| He 2019 | Nicorandil: 59. 43 ± 8. 60  control:  59. 95 ± 8. 40 | NA | NA | NA | Nicorandil:  100. 43 ± 28. 51  control:  105. 39 ± 29. 43 |  | Nicorandil:  138. 81 ± 46. 50  control:  140. 93 ± 30. 22 | PCI | 72 hours | Hydration | Nicorandil:  intravenous nicorandil was administered from 5 h before to 24 h after PCI (42) | Placebo  (43) | Incidence of CIN; the changes in SCr, β2-MG, CysC, eGFR, hs-CRP, SAA, NT-ProBNP, NLR levels before and 48 h, 72 h after surgery |
| Zhang 2021 | NA | Trimetazidine: 70.5  control:  68.1 | NA | Trimetazidine: 40.6  control: 38.3 | NA |  | NA | PCI | 14 days | Hydration | Trimetazidine:  35 mg twice orally (387) | NA  (373) | Incidence of CIN; the changes in Scr, BUN, Cys-C, CrCl within 72hours after use of contrast media. The clinical MAEs, including acute heart failure, malignant ventricular arrhythm-  ia, emergent repeat PCI, acute hemodialysis, renal transplanta-  tion, cerebrovascular events, bleeding or coronary artery bypass grafting after the index procedure, and all-cause morta-  lity occurring during the index hospitalization and within 14 days of follow-up |
| Fu 2021 | Trimetazidine: 76.41 ± 4.27  control:  77.14 ± 4.47 | Trimetazidine: 48.4  control: 50.32 | Trimetazidine: 44.5  control:  46.5 | Trimetazidine: 60.6  control: 53.5 | Trimetazidine: 73.38 ± 13.11  control:  74.37 ± 13.93 |  | Trimetazidine: 140.90 ± 16.49  control:  142.40 ± 14.82 | PCI | 14 days | Hydration | Trimetazidine:  20mg thrice daily orally (155) | NAT  (155) | Incidence of CIN; 72hours after  application of CM; Adverse events during hospitalization and 14-day follow-up, included all-cause mortality, hypotension or severe decrease in blood pressure, acute heart failure, coronary artery bypass, and graft  cerebrovascular events |
| Mirhosseini 2019 | Trimetazidine: 65 ± 6  control:  67 ± 6 | Trimetazidine: 40  control: 48 | Trimetazidine: 64  control: 62 | Trimetazidine: 68  control: 56 | Trimetazidine:  50 ± 7  control:  50 ± 8 |  | Trimetazidine:  116.80(mean)  control: 121.80(mean) | CA/coronary angioplasty | 14 days | Hydration | Trimetazidine:  35 mg twice daily orally (50) | NA  (50) | Incidence of CIN; the changes in serum creatinine; urea concentrations; uNGAL |
| Ye 2017 | Trimetazidine: 63.2 ± 8.3  control:  65.3 ± 8.7 | Trimetazidine: 59.26  control: 59.61 | Trimetazidine: 100  control: 100 | Trimetazidine: 79.63  control: 61.54 | Trimetazidine:  30-89  control:  30-89 |  | NA | CA/ PCI | 72 hours | Hydration | Trimetazidine:  20 mg, three times a day orally at 48 hours before and 24 hours after CAG and/or PCI (54) | NA  (52) | Incidence of CIN; the changes in Scr, cystatin C and eGFR; (MACE) including cardiovasc-  ular death, nonfatal myocardial infarction, target vessel revascu-  larization and heart failure |
| Ibrahim 2017 | Trimetazidine: 64.83 ± 7.36  control:  62.88 ± 8.30 | Trimetazidine: 54  control: 60 | Trimetazidine: 52  control: 62 | Trimetazidine: 60  control: 68 | Trimetazidine:  ＜90  control:  ＜90 |  | Trimetazidine: 112.80 ± 67.52  control: 124.40 ± 40.01 | CA | 72 hours | Hydration | Trimetazidine:  35mg twice daily orally for 72 hours starting 48 hours before the procedure (50) | NA  (50) | Incidence of CIN; |
| Liu 2015 | Trimetazidine: 59.0 ± 11.2  control:  58.3 ± 10.7 | Trimetazidine: 56.5  control: 57.1 | Trimetazidine: 61.3  control: 60 | Trimetazidine: 54.8  control: 54.3 | Trimetazidine: 30-89  control:  30-89 |  | Trimetazidine: 124.94 ± 31.65  control:  119.69 ± 34.28 | CA/ PCI | 12 months | Hydration | Trimetazidine:  thrice daily orally 48 hours before and 24 hours after coronary angiography (62) | NA  (70) | Incidence of CIN; cardiac death, nonfatal myocardial infarction and ischemic stroke end-stage kidney disease, revasculariza-  tion, coronary artery bypass graft surgery, congestive heart failure or pulmonary edema and need for permanent pacing |
| Hadi 2020 | Trimetazidine: 64.4 ± 8.1  control:  64.9 ± 7.7 | Trimetazidine: 52.3  control: 62.2 | Trimetazidine: 100  control: 100 | Trimetazidine: 97.7  control: 95.6 | Trimetazidine: 44.2(mean)  control: 42.82(mean) |  | Trimetazidine: 132.4 ± 44.1  control:  138.6 ± 38.3 | PCI | 24 hours | Hydration | Trimetazidine:  35 mg tablet /twice daily, modified release tablet as a period of three days, starting 48 hours before surgical procedure and for 24 hours post he procedure (44) | NA  (45) | Incidence of CIN; changes in urinary NGAL, HMGB1, NF-kB, TLR 2 |
| Onbasili 2007 | Trimetazidine: 61 ±10  control:  60 ±11 | Trimetazidine: 62.5  control: 76.2 | Trimetazidine: 20  control: 26 | NA | Trimetazidine:  54.4 ± 13  control:  52.4 ± 11 |  | Trimetazidine: 225 (mean)  control:  240(mean) | CA/coronary angioplasty | 7 days | Hydration | Trimetazidine:  20 mg thrice daily orally) for 72 h starting 48 h before the procedure (40) | NA  (42) | Incidence of CIN |

Abbreviations: AKI: acute kidney injury; CA: coronary angiography; CK: creatine kinase; CIN: contrast-induced nephropathy; eGFR: estimated glomerular filtration rate; hs-CRP: high-sensitivity C-reactive protein; HMGB1: High mobility group box-1; MACCE: major adverse cardiac and cerebrovascular events; MACE: Major adverse cardiovascular events; MAEs: major adverse events; NA: not available; NAT: no additional pre-treatment; NGAL: neutrophil gelatinase-associated lipocalin; NLR: neutrophil lymphocyte ratio; PCI: percutaneous coronary intervention; PTCA: percutaneous transluminal coronary angioplasty; PTX3: plasma pentraxin 3; SCr: serum creatinine; SAA: serum amyloid A; TF-TAVI: transfemoral transcatheter aortic valve imp; TLRs: Toll-like receptor.

1. **Quality assessment**

4.1 Quality assessment of the analysis concerned with the incidence of CIN





Figure S1 Risk of bias graph of the analysis concerned with the impact of RIPC, Nicorandil, and trimetazidine on CIN occurrence. Green for low risk, yellow for unclear risk, red for high risk.


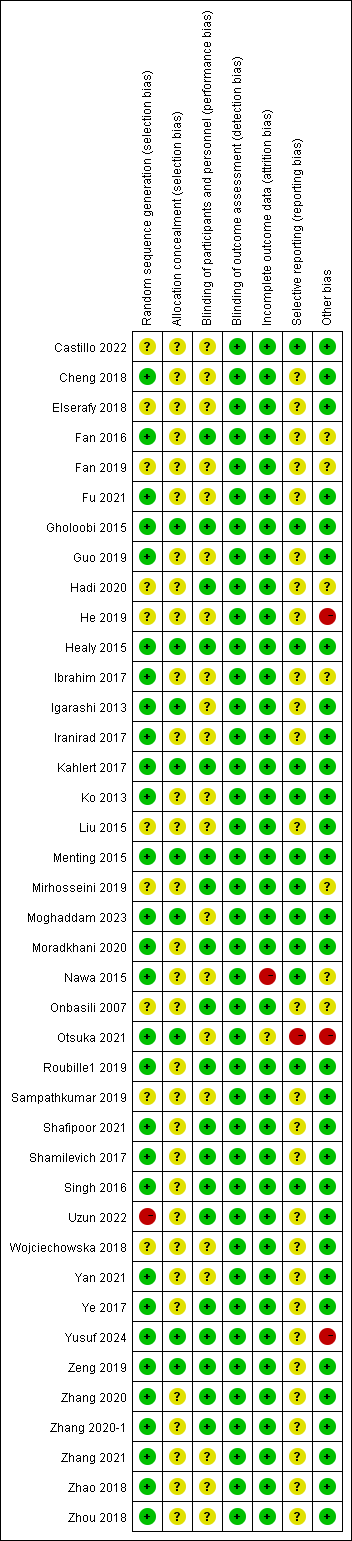


Figure S2 Risk of bias summary of the analysis concerned with the impact of RIPC, Nicorandil, and trimetazidine on CIN occurrence. Green for low risk, yellow for unclear risk, red for high risk.

4.2 Quality assessment of the analysis concerned with the secondary endings: the requirement of hemodialysis, and the incidence of mortality.

4.2.1 The requirement of hemodialysis.


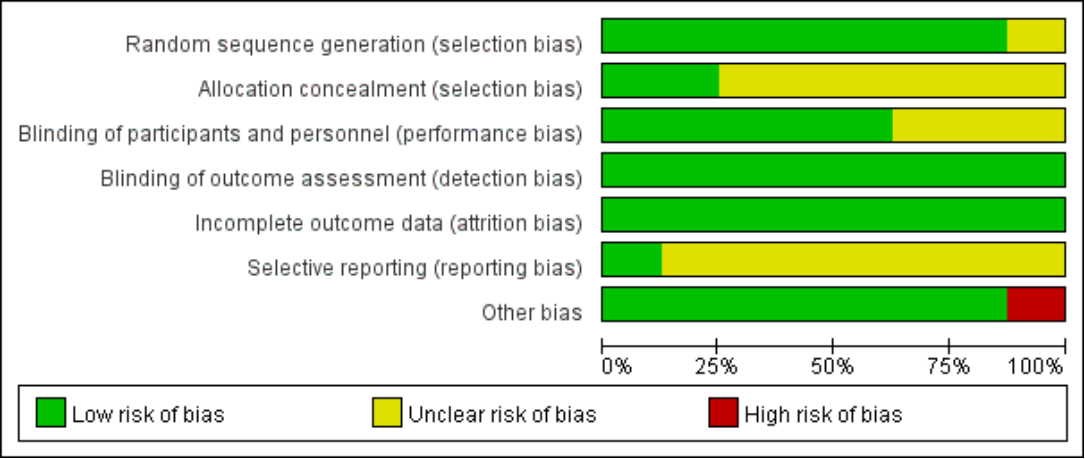


Figure S3 Risk of bias graph of the analysis concerned with the impact of RIPC, Nicorandil, and Trimetazidine on the requirement of hemodialysis.


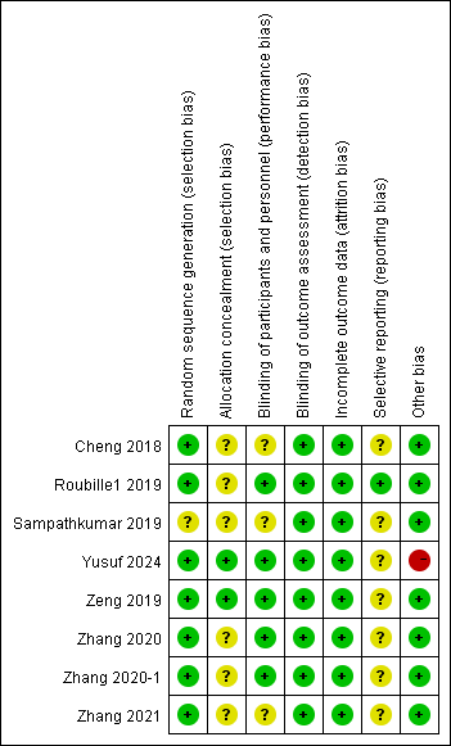


Figure S4 Risk of bias summary graph of the analysis concerned with the impact of RIPC, Nicorandil, and Trimetazidine on the requirement of hemodialysis.

4.2.2 The all-cause mortality


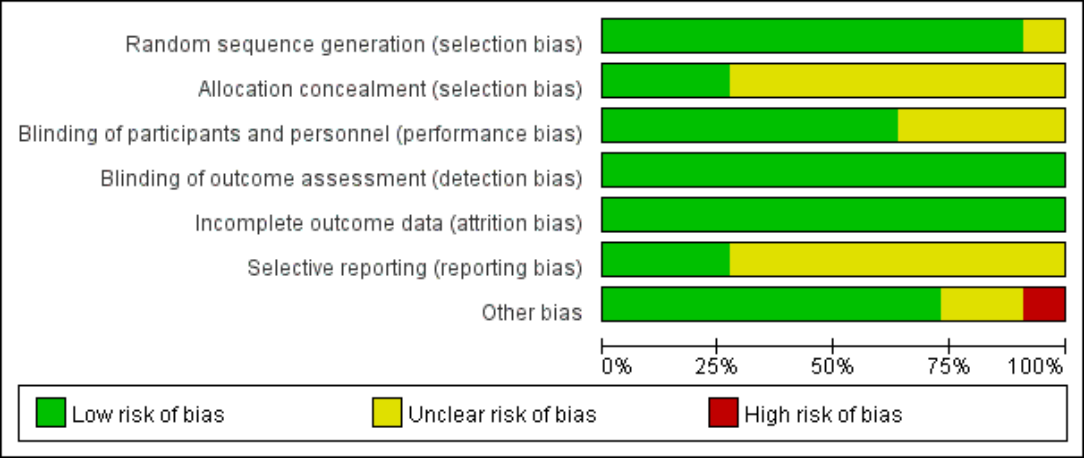


Figure S5 Risk of bias graph of the analysis concerned with the impact of RIPC, Nicorandil, and Trimetazidine on the all-cause mortality.


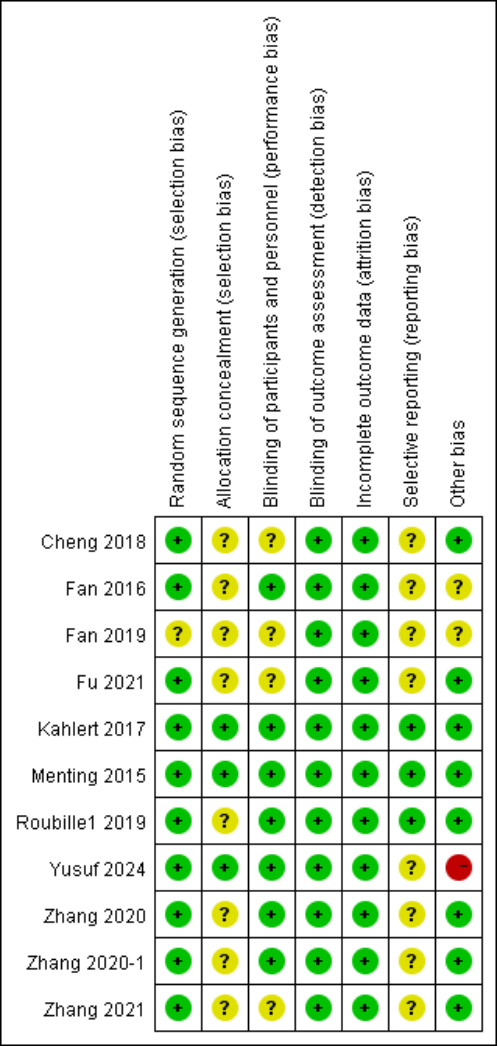


Figure S6 Risk of bias summary graph of the analysis concerned with the impact of RIPC, Nicorandil, and Trimetazidine on the all-cause mortality.

1. **Harbord test**

**Table S2 Harbord test for assessing publication bias of the analysis concerned with the impact of** **RIPC, Nicorandil, and trimetazidine on CIN occurrence**

| Intervention | Z/sqrt(v) | Coefficient | std. err. | t | P>|t| | [95% conf. interval] | | Test of Ho (p) |
| --- | --- | --- | --- | --- | --- | --- | --- | --- |
| RIPC | sqrt(V) | -.1465504 | .5847056 | -0.25 | 0.805 | -1.380171 | 1.087071 | 0.407 |
|  | bias | -.9408858 | 1.107104 | -0.85 | 0.407 | -3.276671 | 1.3949 |  |
| Nicorandil | sqrt(V) | -1.169391 | .4028657 | -2.90 | 0.014 | -2.056093 | -.2826898 | 0.655 |
|  | bias | .3989919 | .87015384 | 0.46 | 0.655 | -1.516204 | 2.314187 |  |
| trimetazidine | sqrt(V) | -.4226353 | .2170922 | -1.95 | 0.099 | -.9538407 | .1085701 | 0.083 |
|  | bias | .9962221 | .4804438 | -2.07 | 0.083 | -2.171826 | .1793815 |  |

1. **Sensitivity analysis**


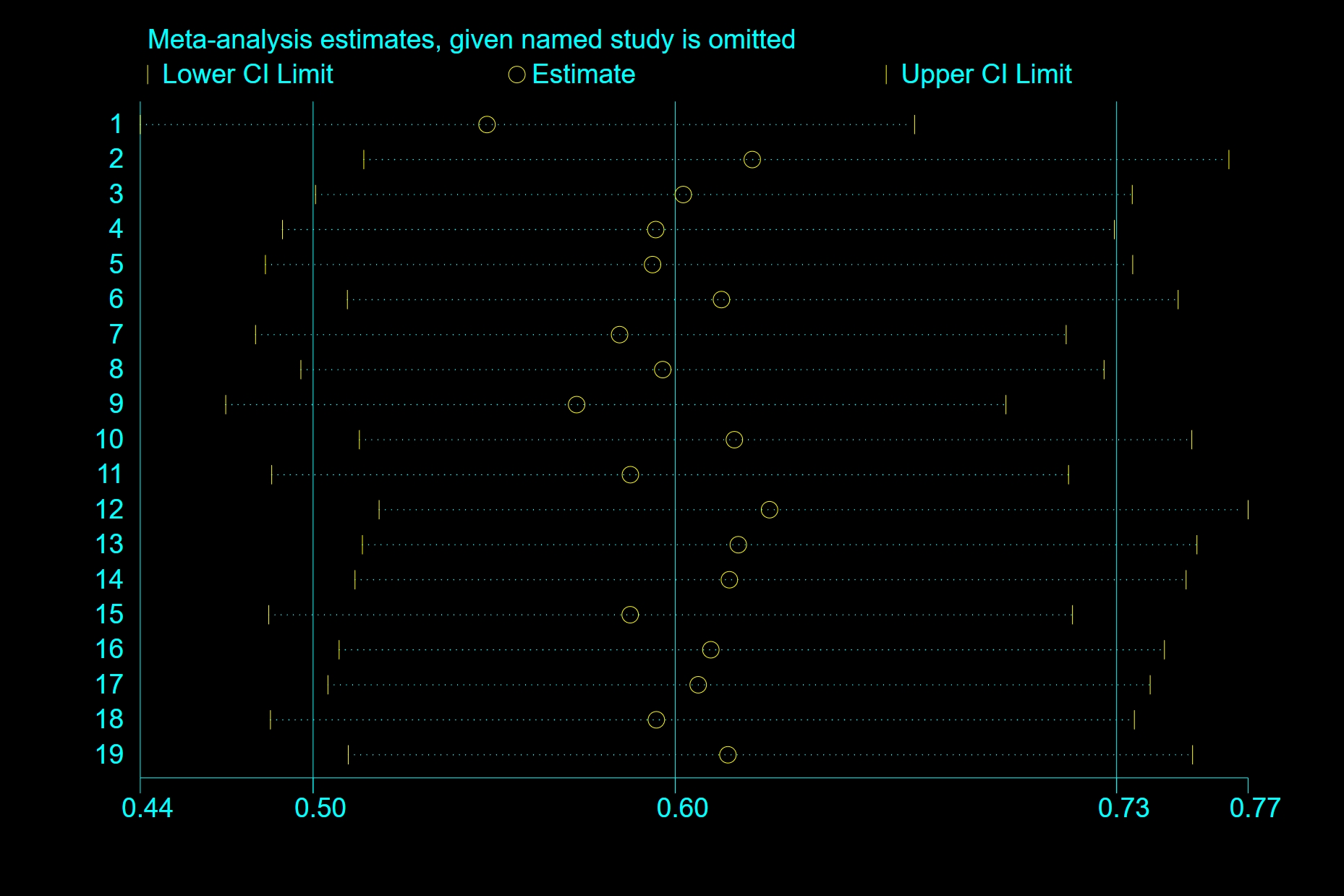


Figure S7 The sensitivity of the analysis concerned with the impact of RIPC on CIN occurrence.


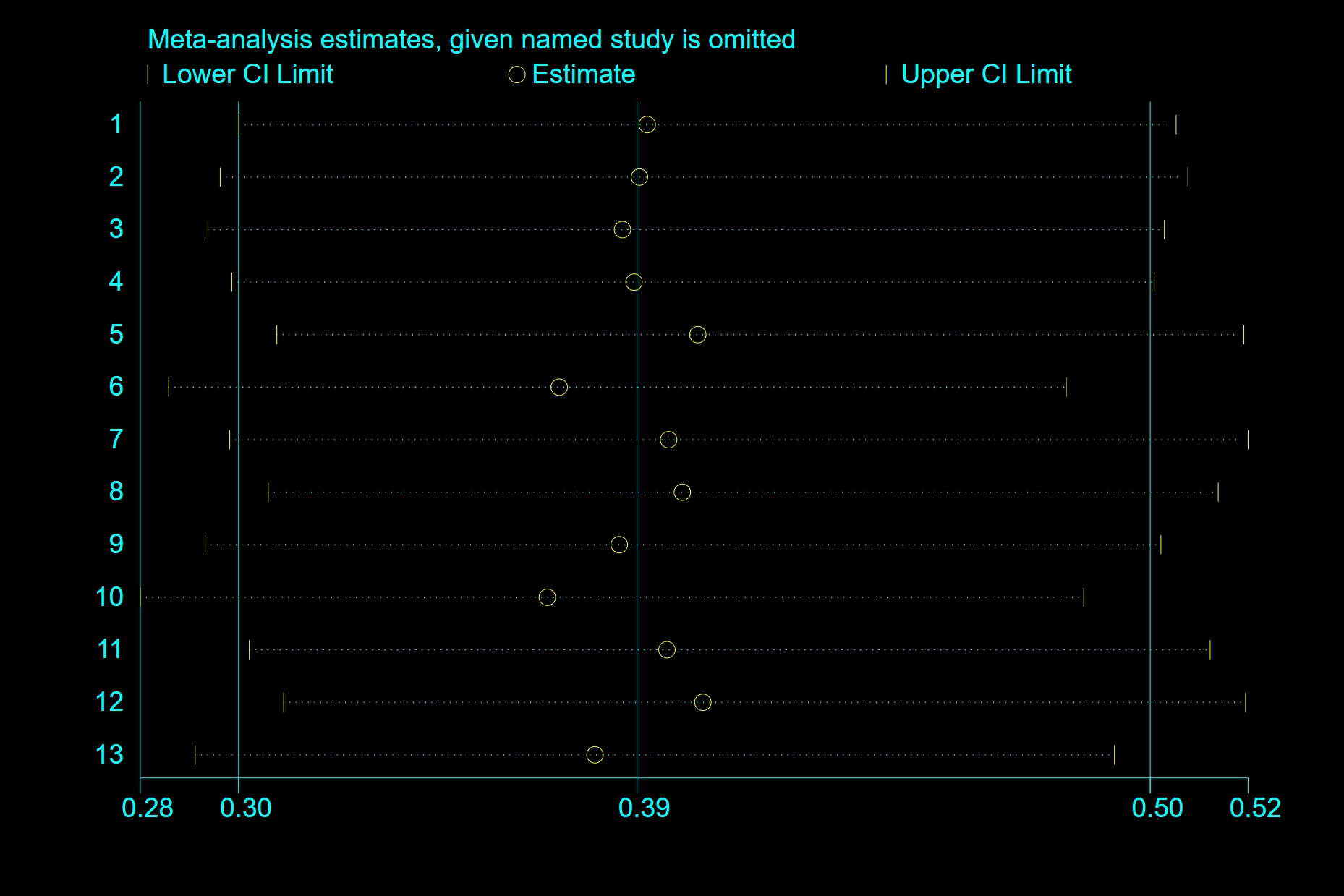


Figure S8 The sensitivity of the analysis of the impact of nicorandil on CIN occurrence.


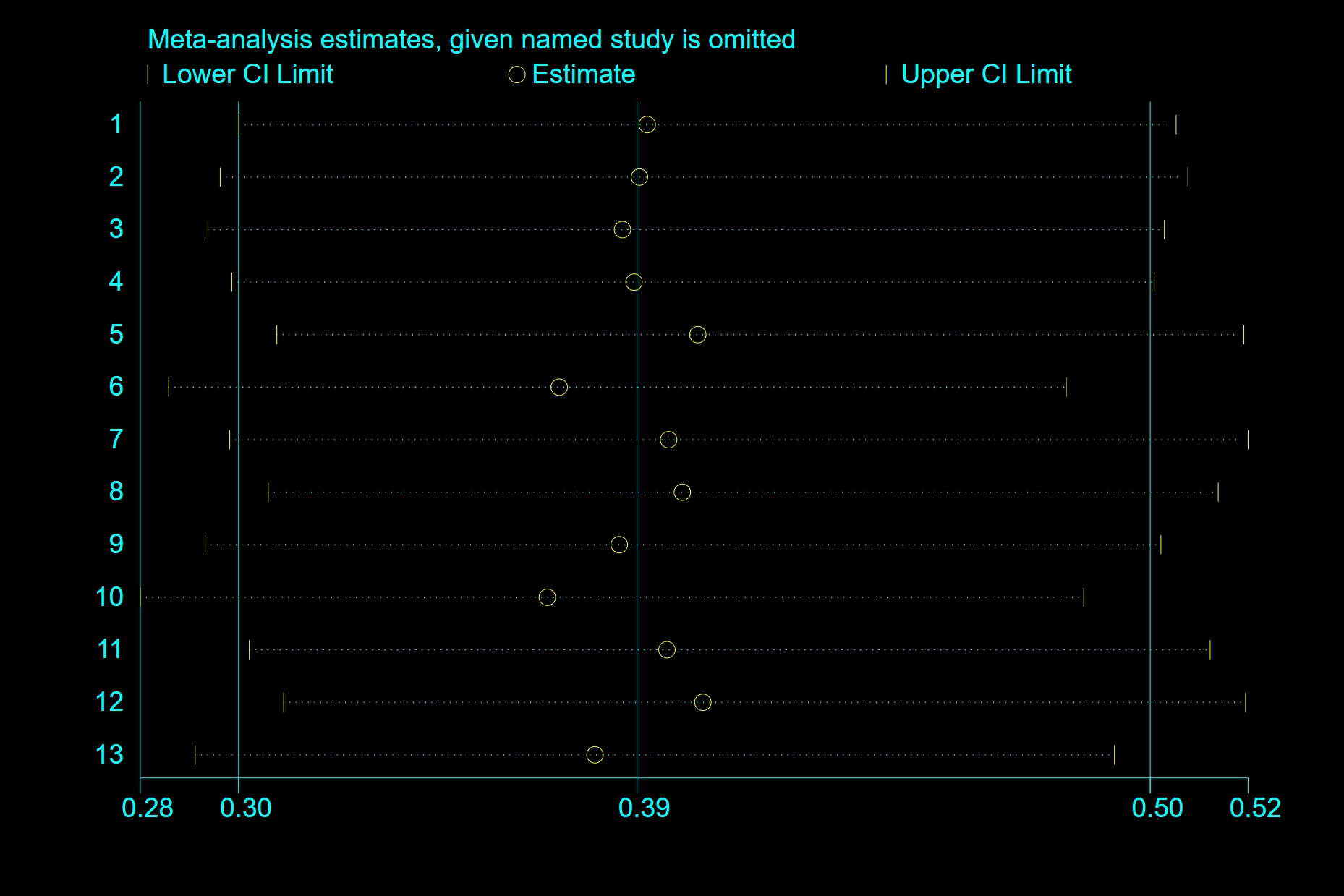


Figure S9 The sensitivity of the analysis of the impact of trimetazidine on CIN occurrence.

1. **Analysis of heterogeneity**

7.1 The heterogeneity of the analysis concerned with the impact of RIPC, Nicorandil, and trimetazidine on CIN occurrence.

**Table S3. The heterogeneity of the subgroup analysis of CIN**

| Per-comparison I-squared | | | | | |  | Global I-squared: | | |
| --- | --- | --- | --- | --- | --- | --- | --- | --- | --- |
|  | t1 | t2 | I^2^.pair | I^2^.cons | Incons.p |  |  | I^2^.pair | I^2^.cons |
| 1 | A | B | 49.14627 | 49.29599 | NA |  | 1 | 18.32141 | 14.61271 |
| 2 | A | C | NA | 0.00000 | 0.7170679 |  |  |  |  |
| 3 | B | C | 0.00000 | 0.00000 | NA |  |  |  |  |
| 4 | B | D | 0.00000 | 0.00000 | NA |  |  |  |  |
| 5 | C | D | NA | 0.00000 | 0.9019647 |  |  |  |  |

A: RIPC; B: Control; C: Nicorandil; D: Trimetazidine.

7.2 The heterogeneity of the subgroup analysis of CIN occurrence among the population with mean eGFR＜60 mL/min/1.73 m^2^.

**Table S4. The heterogeneity of the subgroup analysis of CIN occurrence among the population with mean eGFR＜60 mL/min/1.73 m^2^.**

| Per-comparison I-squared | | | | | |  | Global I-squared: | | |
| --- | --- | --- | --- | --- | --- | --- | --- | --- | --- |
|  | t1 | t2 | I^2^.pair | I^2^.cons | Incons.p |  |  | I^2^.pair | I^2^.cons |
| 1 | A | B | 45.39103 | 45.38759 | NA |  | 1 | 37.26101 | 37.26616 |
| 2 | B | C | 39.70495 | 39.75968 | NA |  |  |  |  |
| 3 | B | D | 0.00000 | 0.00000 | NA |  |  |  |  |

A: RIPC; B: Control; C: Nicorandil; D: Trimetazidine.

7.3 The heterogeneity of subgroup analysis of CIN occurrence among the population with mean eGFR>60 mL/min/1.73 m^2^.

**Table S5. The heterogeneity of subgroup analysis of CIN occurrence among the population with mean eGFR>60 mL/min/1.73 m^2^.**

| Per-comparison I-squared | | | | | |  | Global I-squared: | | |
| --- | --- | --- | --- | --- | --- | --- | --- | --- | --- |
|  | t1 | t2 | I^2^.pair | I^2^.cons | Incons.p |  |  | I^2^.pair | I^2^.cons |
| 1 | A | B | 61.17536 | 61.61556 | NA |  | 1 | 31.51374 | 24.96255 |
| 2 | A | C | NA | 0.00000 | 0.6682330 |  |  |  |  |
| 3 | B | C | 0.00000 | 0.00000 | NA |  |  |  |  |
| 4 | B | D | 0.00000 | 0.00000 | NA |  |  |  |  |
| 5 | C | D | NA | 0.00000 | 0.8427567 |  |  |  |  |

A: RIPC; B: Control; C: Nicorandil; D: Trimetazidine.

7.4 The heterogeneity of the subgroup analysis of CIN occurrence in populations with >50% diabetes.

**Table S6. The heterogeneity of the subgroup analysis of CIN occurrence in populations with >50% diabetes.**

| Per-comparison I-squared | | | | | |  | Global I-squared: | | |
| --- | --- | --- | --- | --- | --- | --- | --- | --- | --- |
|  | t1 | t2 | I^2^.pair | I^2^.cons | Incons.p |  |  | I^2^.pair | I^2^.cons |
| 1 | A | B | 46.66308 | 46.6688 | NA |  | 1 | 0 | 0 |
| 2 | B | C | 0.00000 | 0.0000 | NA |  |  |  |  |
| 3 | B | D | 0.00000 | 0.0000 | NA |  |  |  |  |
| 4 | D | D | NA | 0.0000 | 0.8914503 |  |  |  |  |

A: RIPC; B: Control; C: Nicorandil; D: Trimetazidine.

7.5 The heterogeneity of the subgroup analysis of CIN occurrence in populations

with <50% diabetes.

**Table S7 The heterogeneity of the subgroup analysis of CIN occurrence in populations with <50% diabetes.**

| Per-comparison I-squared | | | | | |  | Global I-squared: | | |
| --- | --- | --- | --- | --- | --- | --- | --- | --- | --- |
|  | t1 | t2 | I^2^.pair | I^2^.cons | Incons.p |  |  | I^2^.pair | I^2^.cons |
| 1 | A | B | 57.000079 | 57.029983 | NA |  | 1 | 45.03921 | 45.15296 |
| 2 | B | C | 4.968257 | 5.625769 | NA |  |  |  |  |
| 3 | B | D | 0.000000 | 0.00000 | NA |  |  |  |  |

A: RIPC; B: Control; C: Nicorandil; D: Trimetazidine.

7.6 The heterogeneity of the analysis concerned with the impact of RIPC, Nicorandil, and Trimetazidine on the requirement of hemodialysis.

**Table S8. The heterogeneity of the analysis concerned with the impact of RIPC, Nicorandil, and Trimetazidine on the requirement of hemodialysis.**

| Per-comparison I-squared | | | | | |  | Global I-squared: | | |
| --- | --- | --- | --- | --- | --- | --- | --- | --- | --- |
|  | t1 | t2 | I^2^.pair | I^2^.cons | Incons.p |  |  | I^2^.pair | I^2^.cons |
| 1 | A | B | 98.67286 | 77.06674 | NA |  | 1 | 92.66101 | 42.24308 |
| 2 | B | C | 35.80114 | 48.33924 | NA |  |  |  |  |
| 3 | B | D | 0.00000 | 0.0000 | NA |  |  |  |  |
| 4 | D | D | NA | 0.00000 | 0.8992894 |  |  |  |  |

Abbreviations: A: RIPC; B: Control; C: Nicorandil; D: Trimetazidine.

7.7 The heterogeneity of the analysis concerned with the impact of RIPC, Nicorandil, and Trimetazidine on the all-cause mortality.

**Table S9. The heterogeneity of the analysis concerned with the impact of RIPC, Nicorandil, and Trimetazidine on the all-cause mortality.**

| Per-comparison I-squared | | | | | |  | Global I-squared: | | |
| --- | --- | --- | --- | --- | --- | --- | --- | --- | --- |
|  | t1 | t2 | I^2^.pair | I^2^.cons | Incons.p |  |  | I^2^.pair | I^2^.cons |
| 1 | A | B | 18.87608 | 12.70625 | NA |  | 1 | 12.42954 | 3.471055 |
| 2 | B | C | 0.00000 | 0.00000 | NA |  |  |  |  |
| 3 | B | D | 43.02853 | 42.23243 | NA |  |  |  |  |
| 4 | D | D | NA | 0.00000 | 0.8202444 |  |  |  |  |

A: RIPC; B: Control; C: Nicorandil; D: Trimetazidine.

1. **Trajectory density graphs**


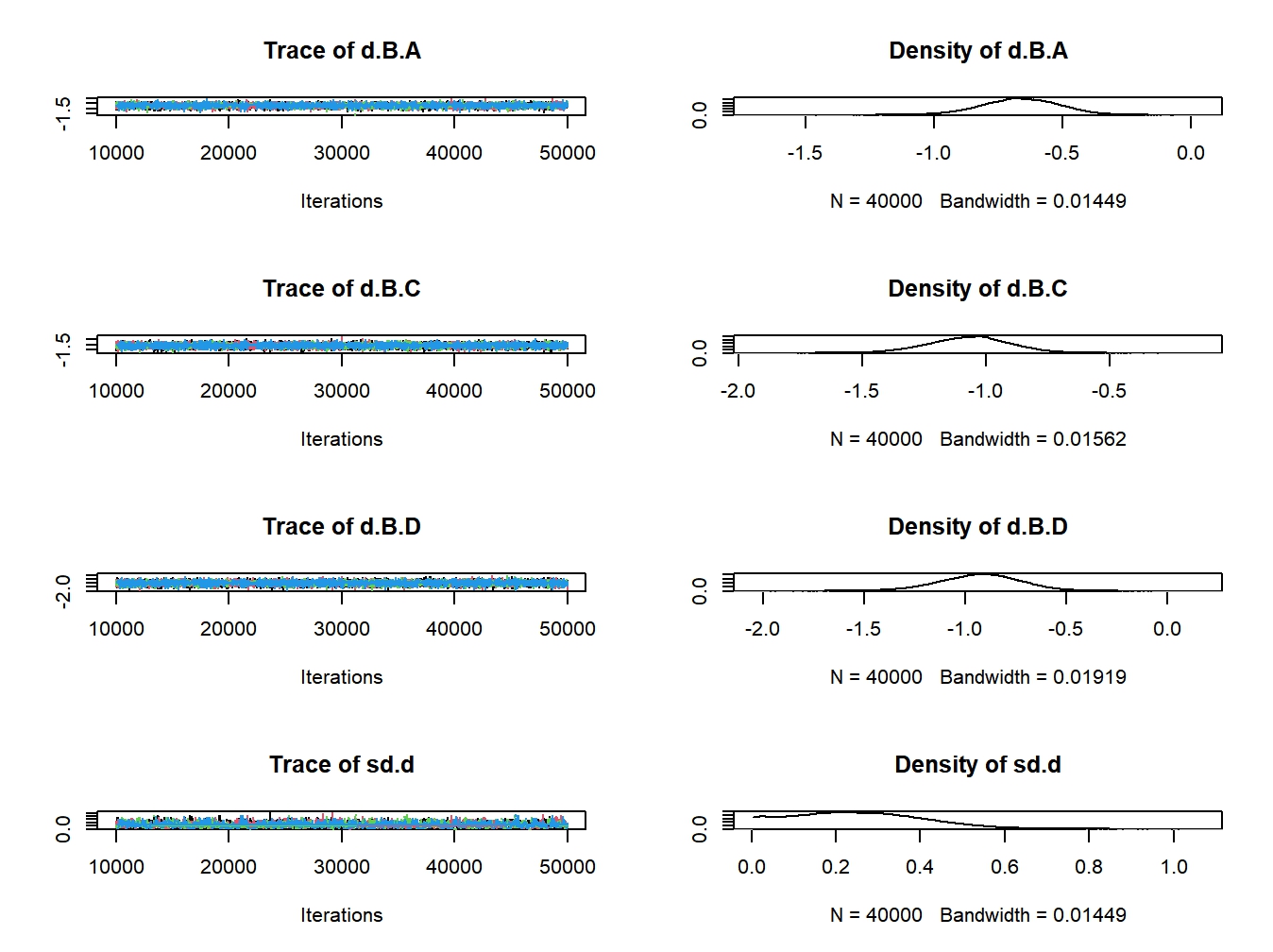


Figure S10 Trajectory density graph of the analysis concerned with the impact of RIPC, Nicorandil, and trimetazidine on CIN occurrence. To assess the model's fit, the more clustered the line segments are, the closer the waveforms are to the graph of a positive distribution or the closer the horizontal coordinates of the wave peaks are to 0, indicating a better fit. A: RIPC; B: Control; C: Nicorandil; D: Trimetazidine


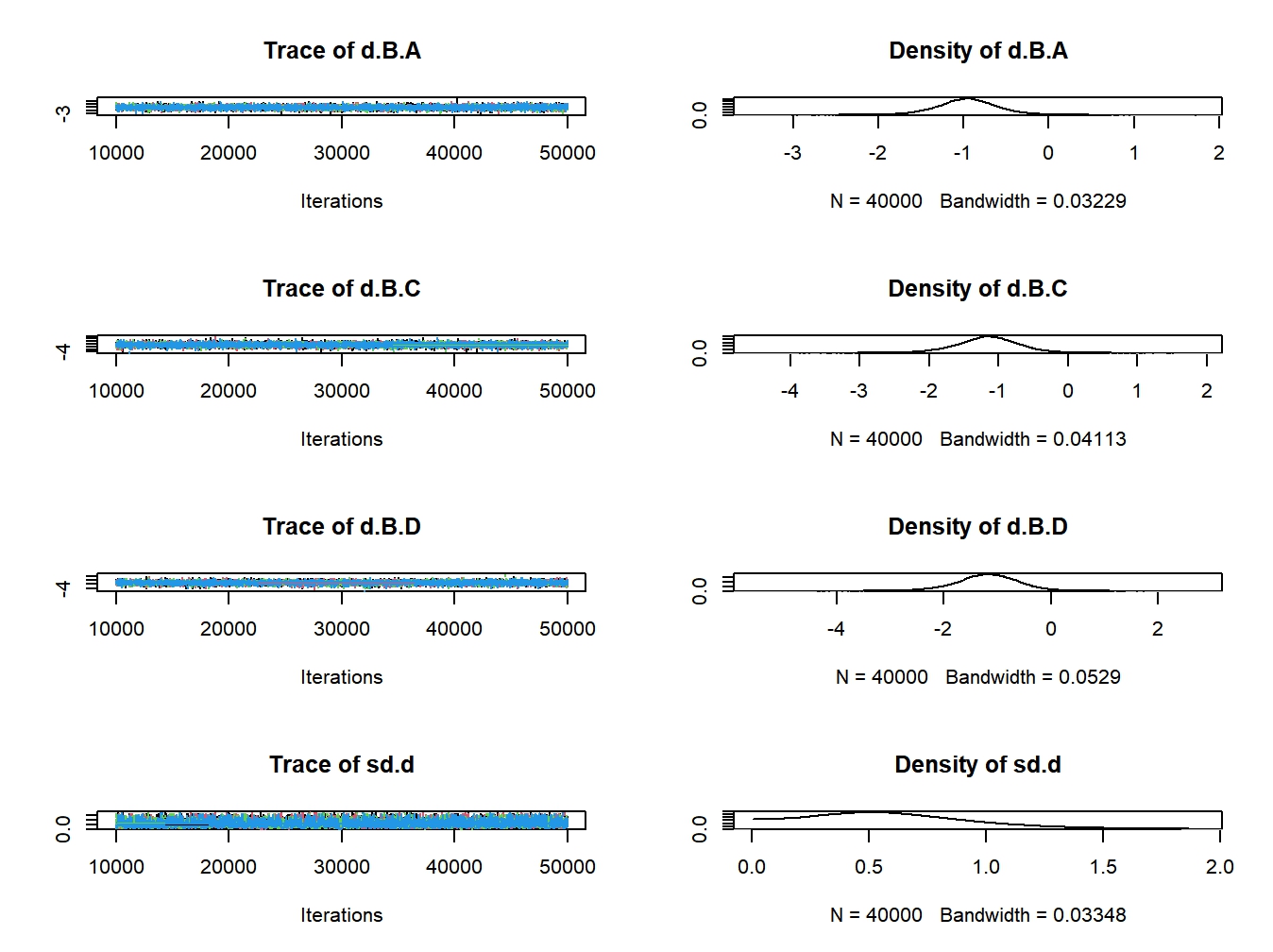


Figure S11 Trajectory density graph of the subgroup analysis of CIN occurrence among the population with mean eGFR<60 mL/min/1.73 m^2^. A: RIPC; B: Control; C: Nicorandil; D: Trimetazidine.


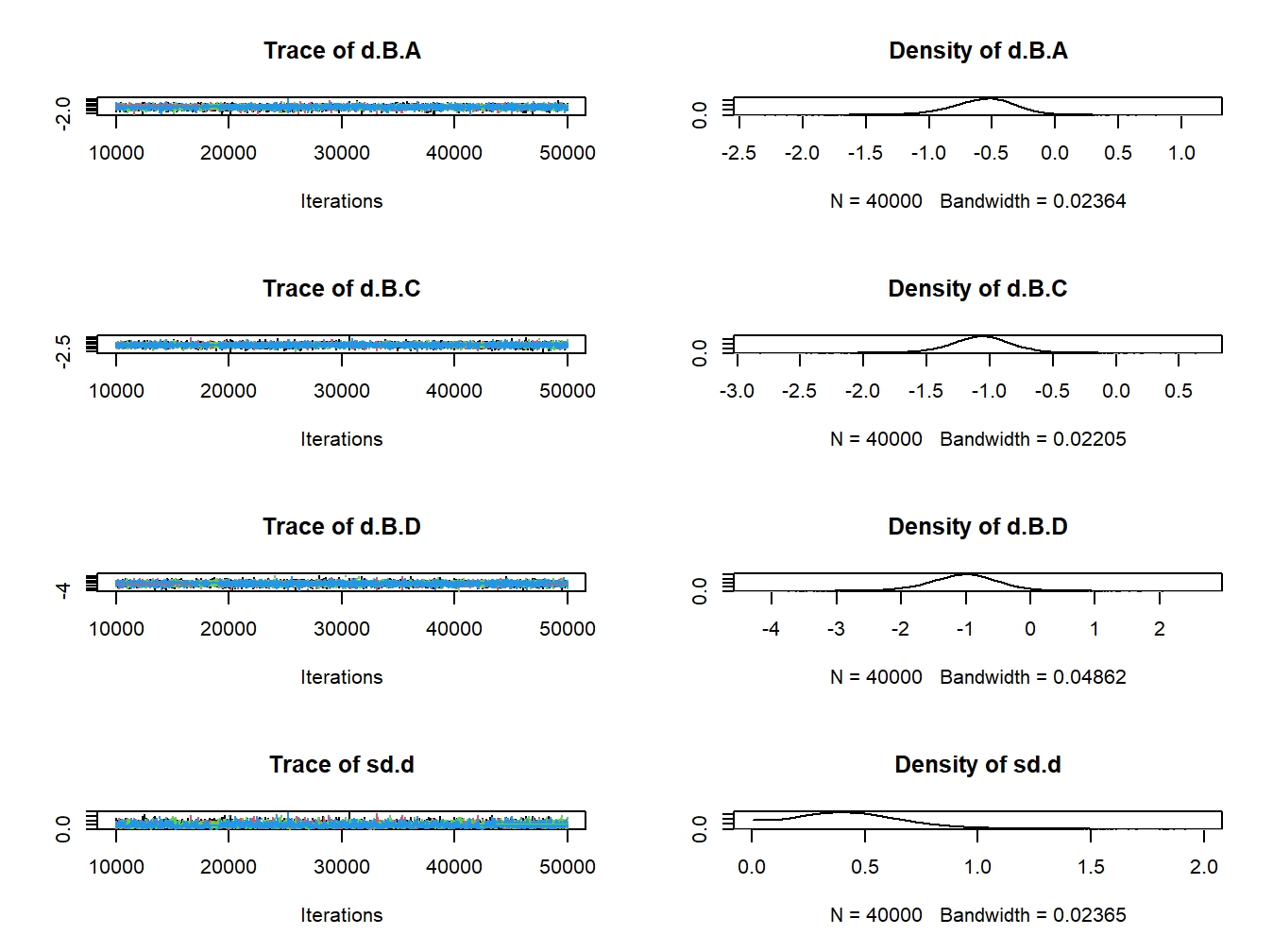


Figure S12 Trajectory density graph of the subgroup analysis of CIN occurrence among the population with mean eGFR>60 mL/min/1.73 m^2^.A: RIPC; B: Control; C: Nicorandil; D: Trimetazidine.


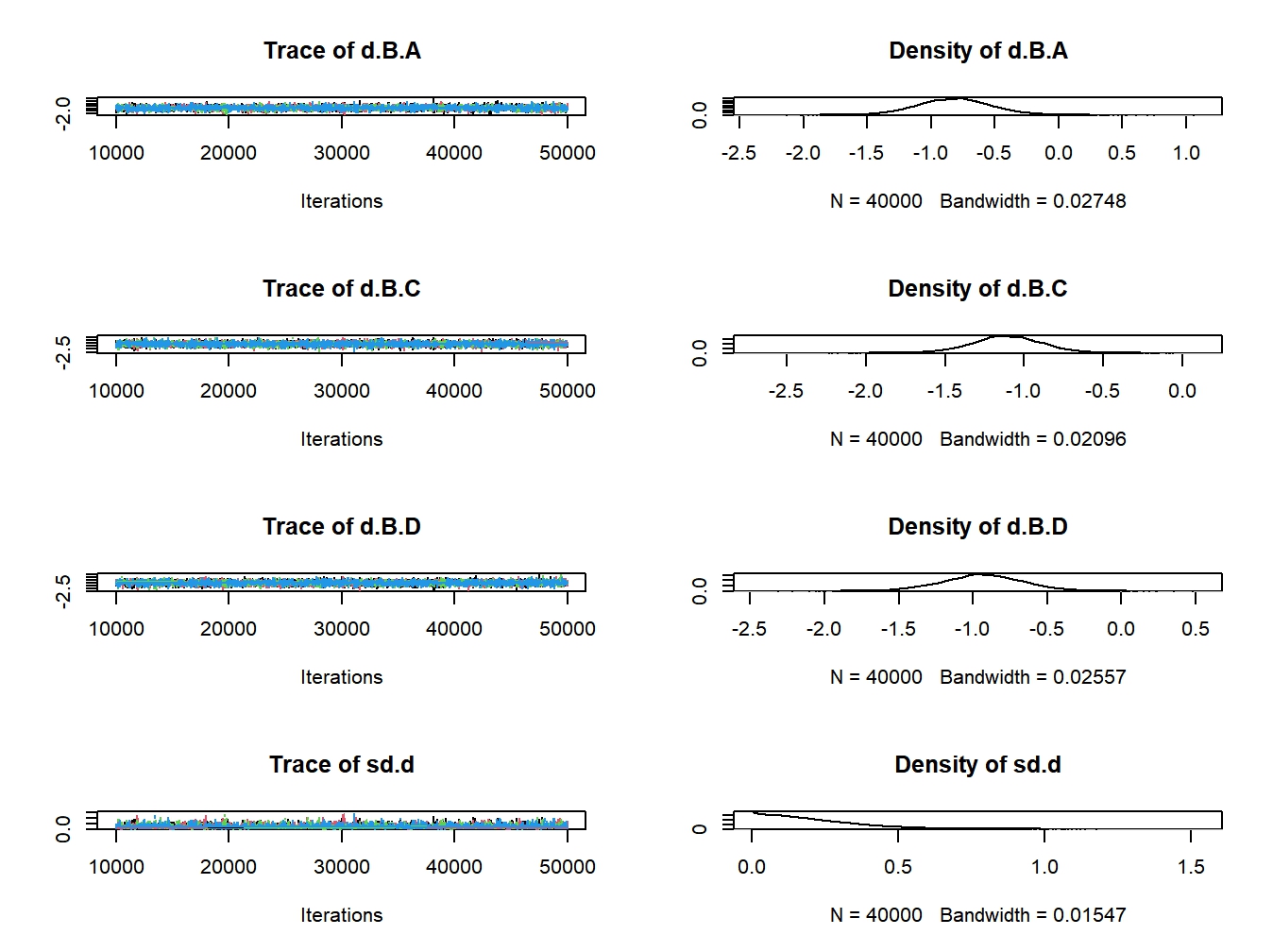


Figure S13 Trajectory density graph of the subgroup analysis of CIN occurrence in populations with>50% diabetes. A: RIPC; B: Control; C: Nicorandil; D: Trimetazidine.


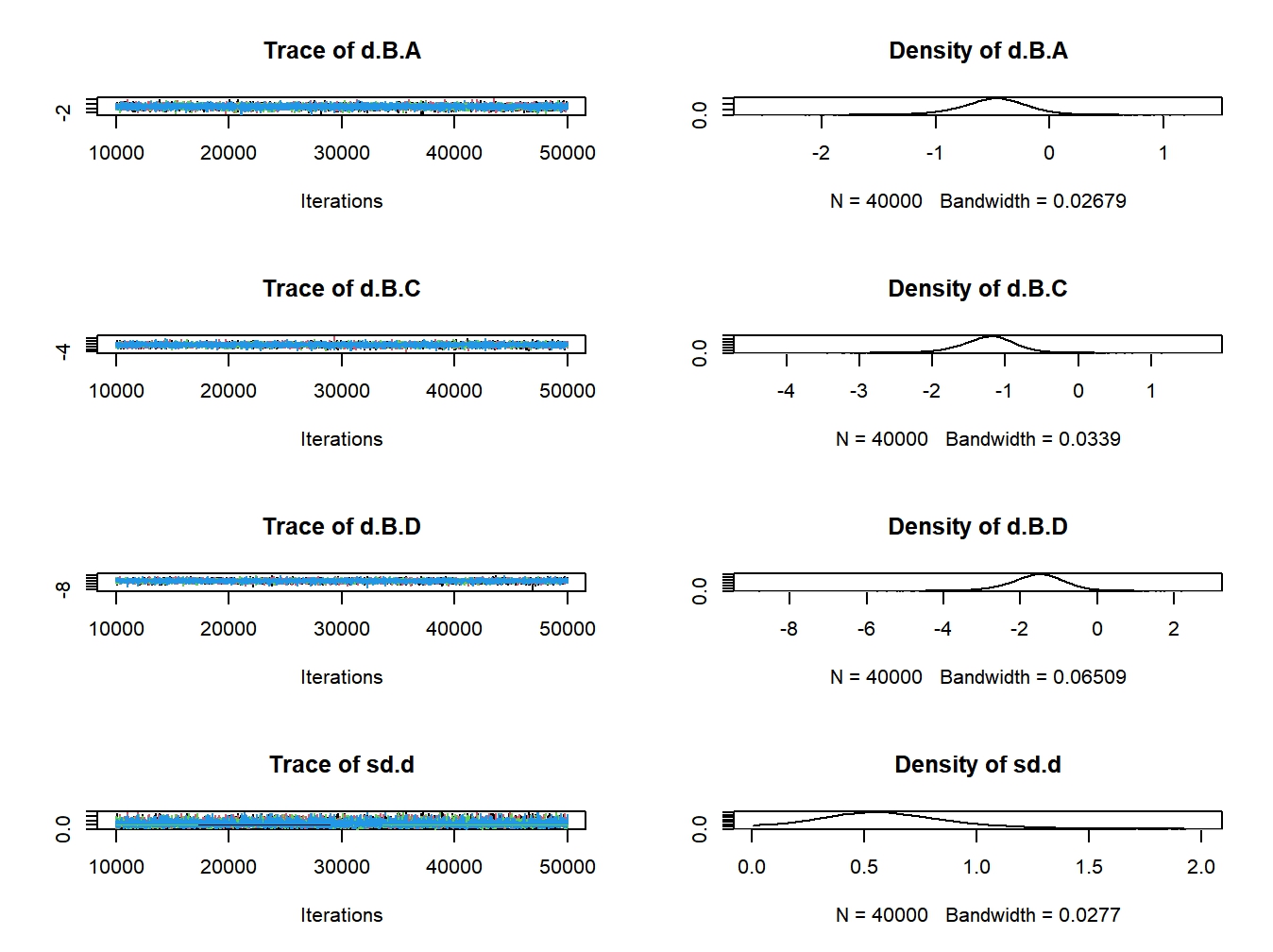


Figure S14 Trajectory density graph of the subgroup analysis of CIN occurrence in populations with <50% diabetes.


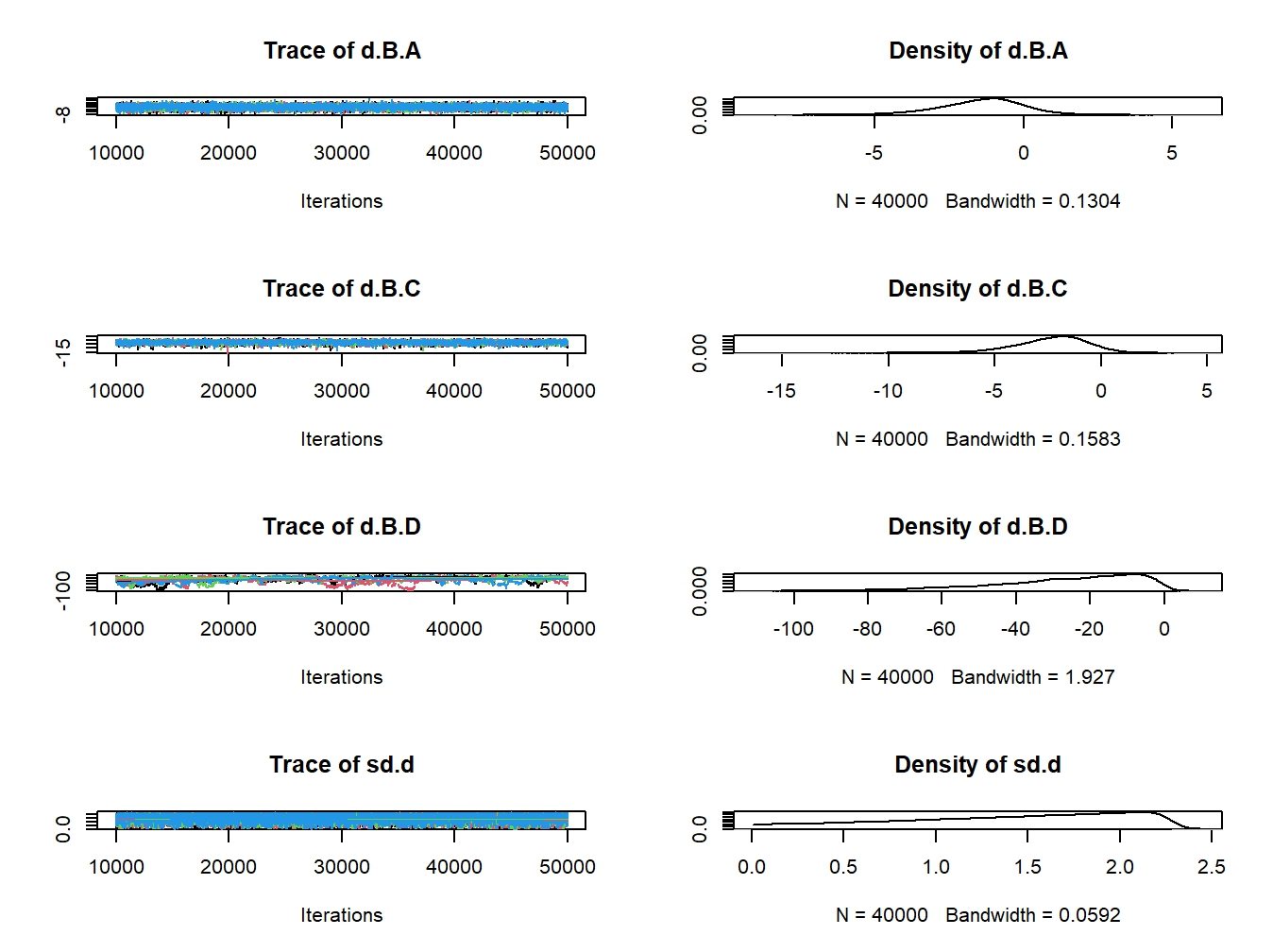


Figure S15 Trajectory density graph of the analysis concerned with the impact of RIPC, Nicorandil, and Trimetazidine on the requirement of hemodialysis. A: RIPC; B: Control; C: Nicorandil; D: Trimetazidine.


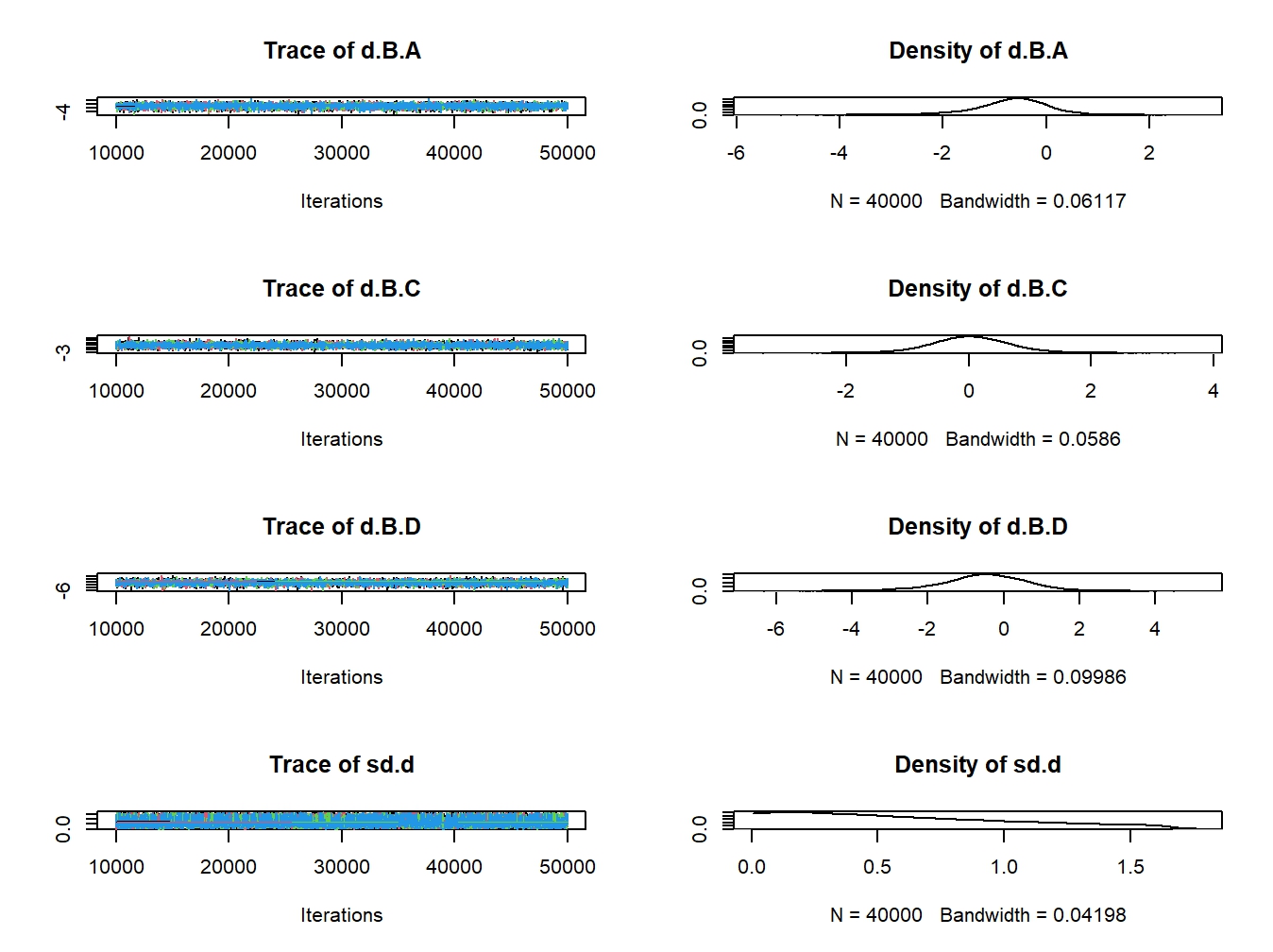


Figure S16 Trajectory density graph of the analysis concerned with the impact of RIPC, Nicorandil, and Trimetazidine on the all-cause mortality. A: RIPC; B: Control; C: Nicorandil; D: Trimetazidine.

1. **The SUCRA results**

**Table S10. The SUCRA results Show the effects of RIPC, Nicorandil, and Trimetazidine in preventing CIN among the population with mean eGFR<60 mL/min/1.73 m^2^.**

| Treatment | SUCRA | PrBest | MeanRank |
| --- | --- | --- | --- |
| RIPC | 58.5 | 19.0 | 2.2 |
| Control | 0.8 | 0.0 | 4.0 |
| Nicorandil | 70.7 | 38.7 | 1.9 |
| Trimetazidine | 70.0 | 42.3 | 1.9 |

**Table S11. The SUCRA results Show the effects of RIPC, Nicorandil, and Trimetazidine in preventing CIN among the population with mean eGFR＞60 mL/min/1.73 m^2^.**

| Treatment | SUCRA | PrBest | MeanRank |
| --- | --- | --- | --- |
| RIPC | 39.3 | 1.0 | 2.8 |
| Control | 0.7 | 0.0 | 4.0 |
| Nicorandil | 84.1 | 54.3 | 1.5 |
| Trimetazidine | 75.9 | 44.7 | 1.7 |

1. **The GRADE results**

**10.1Notes in the GRADE framework**

| ***The risk in the intervention group** (and its 95% confidence interval) is based on the assumed risk in the comparison group and the **relative effect** of the intervention (and its 95% CI).  **CI:** confidence interval; **OR:** odds ratio |
| --- |
| **GRADE Working Group grades of evidence** **High certainty:** we are very confident that the true effect lies close to that of the estimate of the effect. **Moderate certainty:** we are moderately confident in the effect estimate: the true effect is likely to be close to the estimate of the effect, but there is a possibility that it is substantially different. **Low certainty:** our confidence in the effect estimate is limited: the true effect may be substantially different from the estimate of the effect. **Very low certainty:** we have very little confidence in the effect estimate: the true effect is likely to be substantially different from the estimate of effect. |

**10.2 RIPC compared to control for contrast induced nephropathy**

| **Summary of findings:** | | | | | | |
| --- | --- | --- | --- | --- | --- | --- |
| **RIPC compared to control for contrast induced nephropathy** | | | | | | |
| **Patient or population:** contrast induced nephropathy  **Setting:**  **Intervention:** RIPC  **Comparison:** control | | | | | | |
| Outcomes | **Anticipated absolute effects^*^** (95% CI) | | Relative effect (95% CI) | № of participants (studies) | Certainty of the evidence (GRADE) | Comments |
|  | **Risk with control** | **Risk with RIPC** |  |  |  |  |
| CIN incidence | 182 per 1,000 | **104 per 1,000** (74 to 143) | **OR 0.52** (0.36 to 0.75) | 2395 (19 RCTs) | ⨁⨁⨁◯ Moderate^a^ |  |

**Explanations**

a. First, the number of included RCTs was limited. This NMA only included 40 RCTs, and the presence of chance cannot be denied. Further studies can analyze additional treatments and include more studies for comprehensive analysis. Second, there was some heterogeneity in the included studies, such as the surgery patients accepted. Third, hydration therapy was used both in the intervention and control groups. No other drugs reported to have a preventive effect on CIN were used. However, some studies did not mention whether a placebo was used in control groups. This may lead to some bias in the results of the study.

**10.3 Nicorandil compared to control for contrast induced nephropathy**

| **Summary of findings:** | | | | | | |
| --- | --- | --- | --- | --- | --- | --- |
| **Nicorandil compared to control for contrast induced nephropathy** | | | | | | |
| **Patient or population:** contrast induced nephropathy  **Setting:**  **Intervention:** Nicorandil  **Comparison:** control | | | | | | |
| Outcomes | **Anticipated absolute effects^*^** (95% CI) | | Relative effect (95% CI) | № of participants (studies) | Certainty of the evidence (GRADE) | Comments |
|  | **Risk with control** | **Risk with Nicorandil** |  |  |  |  |
| CIN incidence | 133 per 1,000 | **52 per 1,000** (40 to 69) | **OR 0.36** (0.27 to 0.48) | 2964 (13 RCTs) | ⨁⨁⨁◯ Moderate^a^ |  |

**Explanations**

a. First, the number of included RCTs was limited. This NMA only included 40 RCTs, and the presence of chance cannot be denied. Further studies can analyze additional treatments and include more studies for comprehensive analysis. Second, there was some heterogeneity in the included studies, such as the surgery patients accepted. Third, hydration therapy was used both in the intervention and control groups. No other drugs reported to have a preventive effect on CIN were used. However, some studies did not mention whether a placebo was used in control groups. This may lead to some bias in the results of the study.

**10.4 Trimetazidine compared to control for contrast induced nephropathy**

| **Summary of findings:** | | | | | | |
| --- | --- | --- | --- | --- | --- | --- |
| **Trimetazidine compared to control for contrast induced nephropathy** | | | | | | |
| **Patient or population:** contrast induced nephropathy  **Setting:**  **Intervention:** Trimetazidine  **Comparison:** control | | | | | | |
| Outcomes | **Anticipated absolute effects^*^** (95% CI) | | Relative effect (95% CI) | № of participants (studies) | Certainty of the evidence (GRADE) | Comments |
|  | **Risk with control** | **Risk with Trimetazidine** |  |  |  |  |
| CIN incidence | 145 per 1,000 | **66 per 1,000** (48 to 91) | **OR 0.42** (0.30 to 0.59) | 1679 (8 RCTs) | ⨁⨁⨁◯ Moderate^a^ |  |

**Explanations**

a. First, the number of included RCTs was limited. This NMA only included 40 RCTs, and the presence of chance cannot be denied. Further studies can analyze additional treatments and include more studies for comprehensive analysis. Second, there was some heterogeneity in the included studies, such as the surgery patients accepted. Third, hydration therapy was used both in the intervention and control groups. No other drugs reported to have a preventive effect on CIN were used. However, some studies did not mention whether a placebo was used in control groups. This may lead to some bias in the results of the study.
